# Novel HDAC11 inhibitors suppress lung adenocarcinoma stem cell self-renewal and overcome drug resistance by suppressing Sox2

**DOI:** 10.1038/s41598-020-61295-6

**Published:** 2020-03-13

**Authors:** Namrata Bora-Singhal, Durairaj Mohankumar, Biswarup Saha, Christelle M. Colin, Jennifer Y. Lee, Matthew W. Martin, Xiaozhang Zheng, Domenico Coppola, Srikumar Chellappan

**Affiliations:** 1 Department of Tumor Biology, H. Lee Moffitt Cancer Center and Research Institute, 12902 Magnolia Drive, Tampa, FL 33612 USA; 2 FORMA Therapeutics, 500 Arsenal St, Suite 100, Watertown, MA 02472 USA; 3 Department of Anatomic Pathology, H. Lee Moffitt Cancer Center and Research Institute, 12902 Magnolia Drive, Tampa, FL 33612 USA

**Keywords:** Cancer stem cells, Non-small-cell lung cancer

## Abstract

Non-small cell lung cancer (NSCLC) is known to have poor patient outcomes due to development of resistance to chemotherapy agents and the EGFR inhibitors, which results in recurrence of highly aggressive lung tumors. Even with recent success in immunotherapy using the checkpoint inhibitors, additional investigations are essential to identify novel therapeutic strategies for efficacious treatment for NSCLC. Our finding that high levels of histone deacetylase 11 (HDAC11) in human lung tumor tissues correlate with poor patient outcome and that depletion or inhibition of HDAC11 not only significantly reduces self-renewal of cancer stem cells (CSCs) from NSCLC but also decreases Sox2 expression that is essential for maintenance of CSCs, indicates that HDAC11 is a potential target to combat NSCLC. We find that HDAC11 suppresses Sox2 expression through the mediation of Gli1, the Hedgehog pathway transcription factor. In addition, we have used highly selective HDAC11 inhibitors that not only target stemness and adherence independent growth of lung cancer cells but these inhibitors could also efficiently ablate the growth of drug-insensitive stem-like cells as well as therapy resistant lung cancer cells. These inhibitors were found to be efficacious even in presence of cancer associated fibroblasts which have been shown to contribute in therapy resistance. Our study presents a novel role of HDAC11 in lung adenocarcinoma progression and the potential use of highly selective inhibitors of HDAC11 in combating lung cancers.

## Introduction

Lung cancer is the leading cause of cancer related mortality in the US, with about 234,000 new cases expected to be diagnosed in 2018 and 154,000 deaths^1^. Non-small cell lung cancer (NSCLC) accounts for 80–85% of lung cancer and has notably poor survival rates^2^. Early stage NSCLC is treated by surgical resection, radiotherapy or chemotherapy. Chemotherapeutic agents like gemcitabine, platinum compounds and taxanes are widely used to treat both lung adenocarcinomas as well as squamous cell carcinomas, but the success rates vary significantly^3^. NSCLC in smokers generally harbor mutations in the *KRAS* oncogene, while mutations in *EGFR* gene are prevalent in NSCLC in non-smokers. NSCLC has high mutational burden, and hence immunotherapy using checkpoint inhibitors is highly beneficial to a subset of the patients^4,5^. Nevertheless, a significant number of NSCLC patients do not respond to immunotherapy; hence it is imperative to identify novel therapeutic strategies to combat this disease. This notion is further strengthened by the fact that there are no effective drugs that can target KRAS mutant lung cancers; furthermore, while there are highly potent tyrosine kinase inhibitors that target mutant EGFR, patients invariably develop resistance to these inhibitors resulting in recurrence of highly drug resistant metastatic tumors^6,7^.

It has been proposed that cancer stem cells (CSCs) contribute to tumor initiation, dormancy, recurrence and metastasis of various tumors, including NSCLC^8,9^. It has been suggested that eliminating CSCs, in addition to the non-stem cells, is imperative for complete eradication of tumors^10,11^. CSCs are slow dividing cells which can self-renew and are highly drug resistant^12,13^, and thus are refractory to standard chemotherapy drugs and anti-proliferative agents. Embryonic stem cell transcription factors like Oct4, Sox2 and Nanog contribute to the genesis and maintenance of the CSCs^14,15^ and Sox2 is especially important for the self-renewal of stem-like cells from lung adenocarcinomas. Multiple signaling cascades modulate the expression and activity of these transcription factors^11,16^ and our laboratory had shown that the components of the hedgehog signaling pathway and the hippo signaling pathway regulate the expression of Sox2, facilitating the self-renewal of CSCs from lung adenocarcinoma cell lines^17–19^ suggesting that targeting the expression of Sox2 might be a viable approach to eliminate lung adenocarcinoma CSCs. Since transcription factors are difficult to target using small molecule inhibitors, a better approach would be to inhibit molecules that affect their expression or activity. Here we find that novel and highly selective inhibitors of histone deacetylase 11 (HDAC11) might be efficacious in reducing Sox2 expression as well as reducing the viability of NSCLC cells, including CSCs.

The role of histone acetylation has been well studied in chromatin organization and gene regulation^20,21^ and HDAC inhibitors have been approved for clinical use against hematological malignancies as well^22^. HDACs remove acetyl groups from lysine residues on histones, especially histones III and IV in the nucleosome, reducing the access to transcription factors to their target promoters, resulting in transcriptional repression. There are 18 mammalian HDAC family members, which fall into four classes namely class I (HDAC 1, 2, 3 and 8), class II (HDAC 4, 5, 6, 7, 9 and 10), class III (Sirtuins) and class IV which includes only HDAC11^21,23^. HDAC11 is the latest HDAC to be cloned, and its role in normal biology of the cells as well as cancer remains to be fully elucidated.

In the present study, we have shown that HDAC11 is upregulated in cancer stem-like SP cells from NSCLC cell lines. Depletion of HDAC11 reduces Sox2 expression as well as self-renewal of SP cells; additional genes are also affected by depletion of HDAC11. The effects of HDAC11 on the Sox2 promoter were mediated through the Gli1 transcription factor, with which it was found to associate. In addition, novel and highly selective inhibitors of HDAC11 activity can reduce Sox2 expression, eliminate self-renewal and significantly reduce the viability of NSCLC cells and their adherence-independent growth. In addition, these inhibitors are highly effective in eliminating cisplatin-insensitive NSCLC cells as well as cells that are resistant to EGFR inhibitors erlotinib and gefitinib. These inhibitors can selectively eliminate cancer cells even in the presence of primary lung cancer associated fibroblasts (CAFs). These results strongly suggest that inhibiting HDAC11 might  be a viable and practical approach to combat NSCLC, especially lung adenocarcinomas.

## Results

### HDAC11 levels are elevated in lung cancers and it correlates with poor prognosis

**Figure 1:**
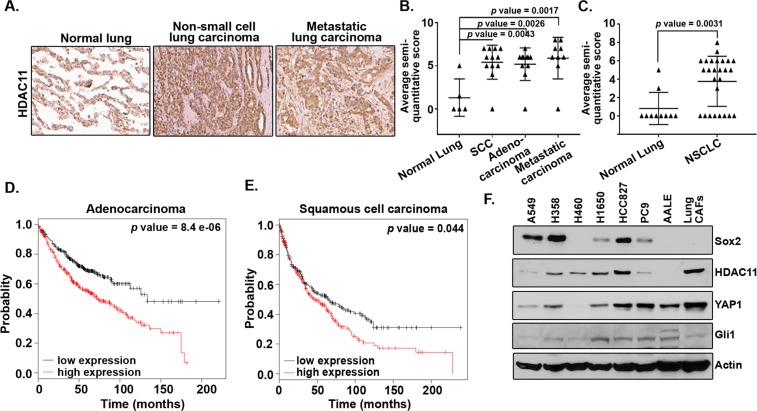
HDAC11 expression in human lung tumor tissues and cells and its correlation with patient prognosis. (**A**) Elevated HDAC11 staining is seen in NSCLC tissue and its metastatic sites as compared to the normal human lung tissue in TMA. (**B**) Quantitation of IHC performed on the TMA shows a ~2.5–3-fold increase in HDAC11 expression in human lung adenocarcinoma tissue, squamous cell carcinoma (SCC) and metastatic lung carcinoma as compared to normal lung tissue. (**C**) Quantitation of second IHC staining for HDAC11 also showed 3-fold increase in HDAC11 expression in NSCLC patient tumor tissue as compared to normal lung tissue. (**D**,**E**) Kaplan-Meier survival analysis for HDAC11 (Probeset 219847_at) shows poor prognosis in lung adenocarcinoma (**D**) and squamous cell carcinoma (**E**) patients with higher mRNA expression of HDAC11 in them. (**F**) HDAC11 could be detected in multiple cell lines of different histology by a western blot analysis. Similarly, Sox2 and YAP1 could also be detected in these cells. The images of the full scan western blots are provided in Supplementary Fig. 6.

Experiments were conducted to assess the potential role of HDAC11 in NSCLC. An immunohistochemical analysis was carried out on human tissue microarrays to assess whether the levels of HDAC11 were altered in lung cancer. We found that the HDAC11 levels were elevated by ~2.5 fold in the lung adenocarcinoma as well as in squamous cell carcinoma as compared to normal lung tissue (Fig. 1A). Quantitation of the staining showed a significant (3 fold) increase in the expression of HDAC11 in metastatic NSCLC of both histological subtypes (Fig. 1B). The HDAC11 staining was also conducted on a second human tissue microarray. The quantitation of the HDAC11 staining in this TMA also showed a significant (3–4 fold) increase in HDAC11 expression in NSCLC tissues as compared to normal lung (Fig. 1C). Since we found an increase in HDAC11 expression in lung cancer, we examined whether the expression of HDAC11 predicts the prognosis of lung cancer patients. A Kaplan Meier survival analysis conducted on the publicly available KM Plotter dataset^24^ (http://kmplot.com/analysis/index.php?p=service&cancer=lung) using Probeset 219847_at showed that higher expression of HDAC11 correlated with poor survival in patients with lung adenocarcinoma as well as squamous cell carcinoma (Fig. 1D,E). Additional multivariate analysis of the data showed poor survival in early stage (Stage 1) lung adenocarcinoma patients with higher expression of HDAC11 (Supplementary Fig. 1A). Also, it was found that higher expression of HDAC11 in male lung adenocarcinoma patients correlated with poor survival (Supplementary Fig. 1B). Such a correlation was not observed in the female patients (data not shown); the reason for this disparity is not clear at this time. While this data was not evident when another probe set was tested, analysis of the Cancer Cell Line Encyclopedia showed that HDAC11 is overexpressed or amplified in 8% of cancer cell lines (data not shown). Similarly, an analysis of the Oncomine database suggested that there are variations in the levels of HDAC11 in tumors indicating that it might depend on the tumor histology or grade of the tumors.

The expression pattern of HDAC11 in lung cancer cell lines was next examined. Western blot analysis was conducted on lysates from lung adenocarcinoma cells, immortalized tracheobronchial cells (AALE) and primary lung cancer associated fibroblasts (CAFs) (Fig. 1F). A549, H358 and H460 cells harbor a *Kras* mutation and H1650, HCC827 and PC9 cells carry mutation in the *Egfr* gene. Sox2, which is known to promote stemness of lung adenocarcinoma and is often amplified in squamous cell carcinoma, was expressed in these cells to varying levels^15,18,25,26^. YAP1, a transcriptional co-activator and the effector protein of hippo signaling pathway^18,27^, was expressed in these cell lines as well (Fig. 1F). Next, we assessed if expression of HDAC11 correlated with the expression of Sox2 in the tissues of lung cancer patients. Immunohistochemical analysis was carried out for HDAC11 and Sox2 on human lung TMAs (Supplementary Fig. 1A,B). HDAC11 expression was higher as observed earlier (Supplementary Fig. 1C). Our earlier publications have shown higher expression of Sox2 in NSCLC patient tumors^17–19^. The results here showed similar higher expression of Sox2 in NSCLC patients (Supplementary Fig. 1D). Pearson correlation coefficient analysis of the semi quantitative scores of HDAC11 and Sox2 showed a moderately positive correlation (*r* = 0.5041) (Supplementary Fig. 1E). These results suggested that HDAC11 was expressed in lung cancer tissues and cell lines and elevated levels of this protein might contribute to poor patient survival.

### HDAC11 is elevated in cancer stem-like cells from lung adenocarcinoma cell lines and regulates Sox2

**Figure 2:**
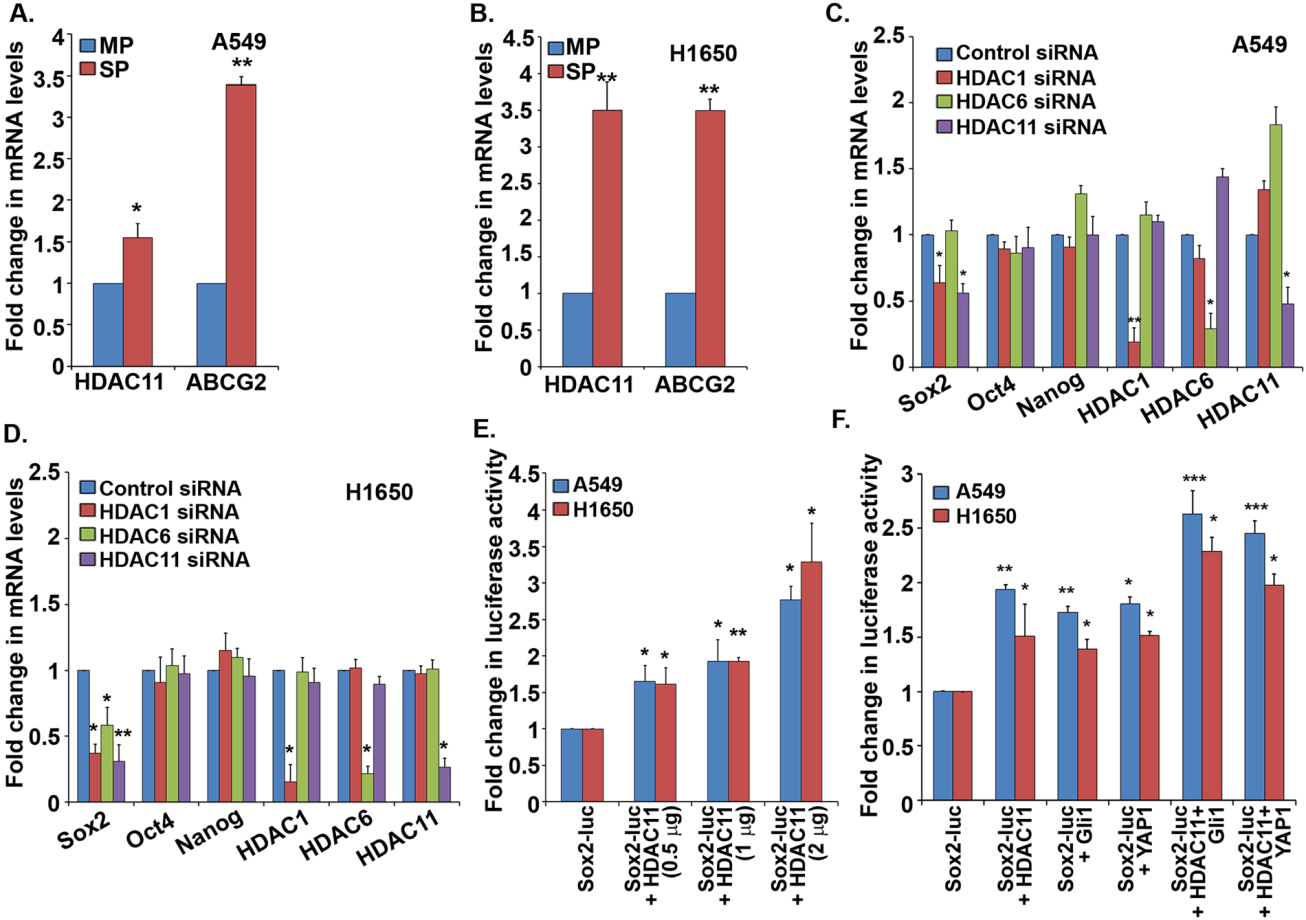
HDAC11 is elevated in stem-like side population (SP) from NSCLC cell lines and regulates Sox2. (**A**,**B**) Real time PCR analysis of mRNA from SP and MP cells of A549 (**A**) and H1650 (**B**) cell lines reveal significantly higher levels of *HDAC11* mRNA in sorted SP cells. *ABCG2* mRNA expression is used as positive control for SP cells. (**C**,**D**) Real time PCR analysis of cells depleted of *HDAC1*, *HDAC6* or *HDAC11* using siRNA transfections in A549 (**C**) and H1650 (**D**) cells show decrease in *Sox2* mRNA expression as compared to control siRNA treatment. There was no change in *Oct4* and *Nanog* expression. *HDAC1*, *HDAC6* and *HDAC11* mRNA expression confirmed the depletion status in both cell lines. (**E**) Transient transfection experiments in A549 and H1650 cells with *Sox2 core promoter* luciferase (Sox2-luc) construct and increasing concentrations of HDAC11 shows increasing promoter luciferase activity. (**F**) Co-transfection experiments of Sox2-luc and HDAC11 with YAP1 and Gli1 expression vectors showed an additive effect on Sox2-luc activity in both A549 and H1650 cells.

Recent reports suggest a possible role of HDACs in maintenance of CSCs, opening a new avenue to target these cells^28^. Since no information is available on the expression of HDACs in stem-like cells from NSCLC, we examined the levels of HDAC11 in Hoechst negative stem-like side population (SP) of NSCLC cell lines. We had found that the SP cells isolated from lung tumor explants as well as NSCLC cell lines have stem-like properties. SP cells could self-renew, were drug resistant and could transdifferentiate into CD31-positive angiogenic tubules^17,18^. A RT-PCR analysis showed that there was 1.5 to 3.5-fold increased expression of *HDAC11* mRNAs in SP cells from A549 (Fig. 2A) and H1650 (Fig. 2B) cell lines, as compared to the non-stem main population (MP) cells. SP cells were found to have higher expression of *ABCG*2 mRNA, which was used as a control (Fig. 2A,B)^17,18^. Since we had found that multiple embryonic stem cell transcription factors are elevated in stem-like SP cells, we examined if HDAC11 regulates the expression of any of these factors. Towards this purpose, we depleted *HDAC11* by siRNAs in A549 and H1650 cells and analyzed the expression of stem cell transcription factors *Sox2*, *Oct4* and *Nanog*. Additional HDACs like *HDAC1* and *HDAC6* were also depleted as they were shown to have a role in self-renewal in embryonic stem cells and regulation of Sox2 in cancer respectively^29,30^. Depletion of *HDAC1* or *HDAC11* resulted in a reduction in the level of *Sox2* mRNA in both A549 and H1650 cells (Fig. 2C,D respectively). However, there was no reduction in the expression of *Oct4* and *Nanog* transcription factors upon the depletion of *HDAC1*, *HDAC6* or *HDAC11* (Fig. 2C,D). The depletion of *HDAC1*, *HDAC6* and *HDAC11* genes was also confirmed by RT-PCR in A549 and H1650 (Fig. 2C,D).

We then investigated if HDAC11 could induce the *Sox2* promoter activity using transient transfection assays. The results showed that *Sox2* promoter luciferase (Sox2-luc) activity increased in a dose dependent manner with increasing amounts of HDAC11 (Fig. 2E). Our earlier studies had shown that the hippo signaling pathway effector protein YAP1 and the hedgehog pathway transcription factor Gli1 could induce *Sox2* transcription^18,19^; experiments were conducted to assess if HDAC11 could enhance the induction of Sox2-luc by YAP1 and Gli1. Transient transfections revealed that co-transfection of HDAC11 had an additive effect on the induction of Sox2-luc promoter by YAP1 or Gli1 (Fig. 2F). These results suggested that HDAC11 might act as downstream effector of the hippo or the hedgehog signaling cascades to induce genes involved in stemness.

### HDAC11 interacts with Gli1 and regulate Sox2 expression

**Figure 3:**
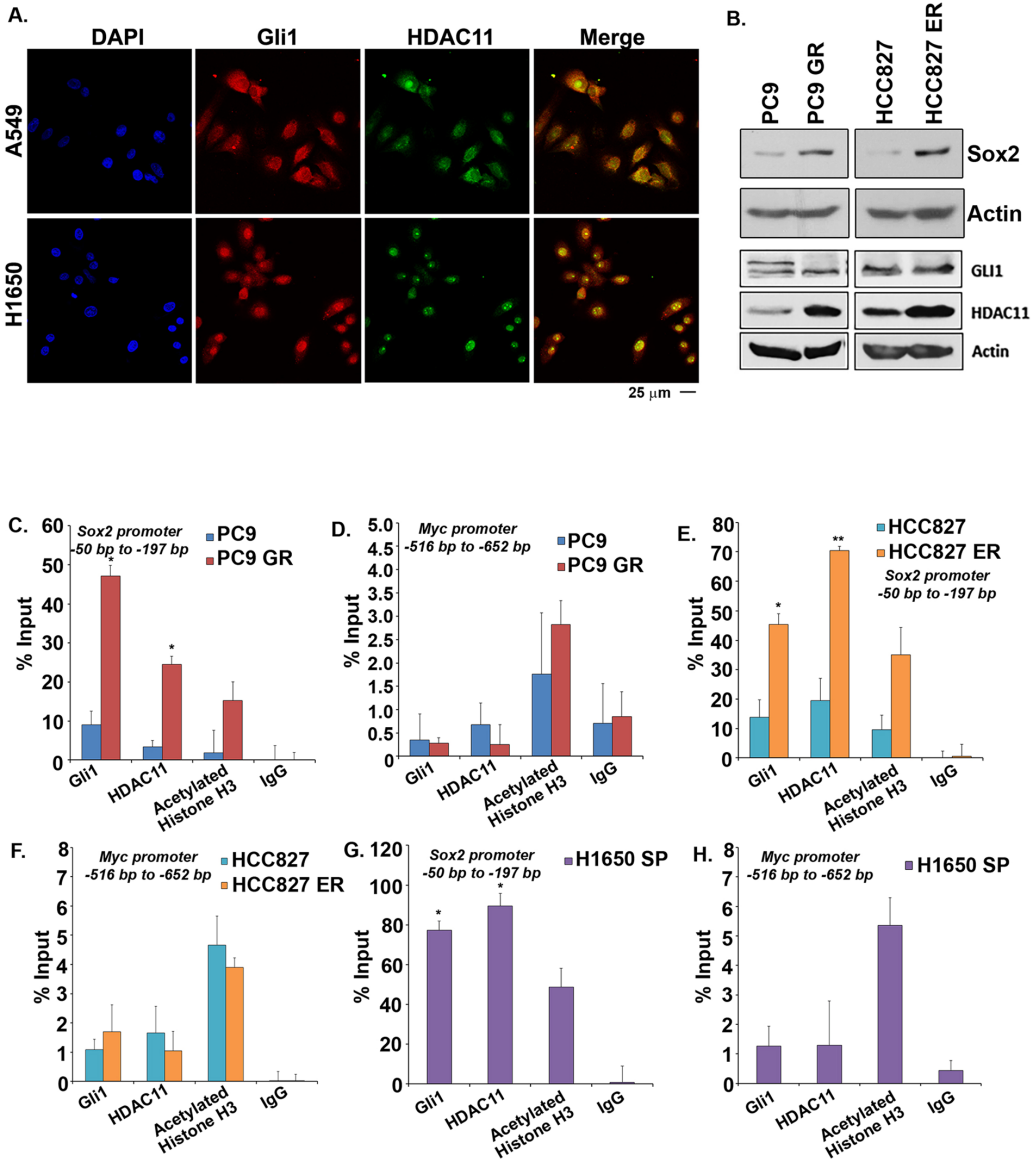
Sox2 gene expression is regulated by Gli1 and HDAC11. (**A**) Double immunofluorescence assay with Gli1 and HDAC11 showed co-localization of both in A549 (upper panel) and H1650 (lower panel) cells. DAPI was used to stain the nucleus of the cells (**B**) Western blot analysis with PC9 parental and gefitinib resistant PC9 GR cells showed an increase in Sox2 and HDAC11 expression in the resistant cells. Increased Sox2 as well as HDAC11 proteins were also found in HCC827 erlotinib resistant (ER) cells as compared to the HCC827 parental cells. (**C**,**D**) A ChIP analysis in PC9/PC9 GR cells showed presence of HDAC11 with Gli1 binding site on Sox2 promoter (**C**) and this interaction was higher at the promoter in the resistant cells. Such a binding was not seen in the Myc promoter (**D**). (**E**,**F**) Similar ChIP analysis in HCC827/HCC827 ER cells showed increased association of HDAC11 with Gli1 at the Sox2 promoter in the erlotinib resistant HCC827 cells (**E**). No change was observed in the Myc promoter that was used as a control. (**G**,**H**) The ChIP analysis on H1650 side population cells showed the interaction of HDAC11 on the Sox2 promoter through the Gli1 binding site (**G**). Myc was used as a control (**H**). The ChIP qPCR was analyzed using two-way ANOVA test; *p < 0.05, **p < 0.01.

As our results showed that HDAC11 could induce *Sox2* promoter activity along with Gli1 transcription factor, we examined if HDAC11 interacted with Gli1 using double immunofluorescence assays. We found that Gli1 and HDAC11 co-localized in both A549 and H1650 cells (Fig. 3A), suggesting that their physical interaction contributes to the induction of Sox2. This result was confirmed by an immunoprecipitation-western blot experiment, which showed the presence of Gli1 in HDAC11 immunoprecipitates (Supplementary Fig. 2). Our earlier report showed that drug resistance in cells lead to increased Sox2 protein expression^19^. Western blot analysis confirmed that higher Sox2 expression in gefitinib resistant PC9-GR cells as compared to parental PC9 cells (Fig. 3B). Similar results were also obtained in erlotinib resistant HCC827-ER cells as compared to parental HCC827 cells (Fig. 3B). Both the resistant cells also had higher levels of HDAC11, while there was no marked change in the levels of Gli1 (Fig. 3B). We next used these cells in chromatin immunoprecipitation (ChIP) assays and found that HDAC11 could be detected on the *Sox2* gene promoter at the Gli1 transcription factor binding sites^19^ (Fig. 3C,E). The interaction was found to be higher in the resistant cells as compared to the parental cells in both HCC827/HCC827 ER and PC9/PC9 GR pairs. Acetylated histone H3 was used as positive control and IgG was used as negative control in this experiment (Fig. 3C,E). *Myc* promoter was used as overall negative control (Fig. 3D,F). Since Sox2 plays a significant role in the maintenance and survival of CSCs from lung adenocarcinoma cells^17–19^, we carried out the ChIP assays in stem-like side population (SP) cells isolated from H1650 lung adenocarcinoma cells. Our results showed presence of HDAC11 on *Sox2* promoter region on Gli1 binding sites in the H1650 SP cells (Fig. 3G). *Myc* was used as negative control here as well (Fig. 3H). Taken together, these results suggest that HDAC11, in association with Gli1, associates with the *Sox2* promoter to induce its expression.

### Depletion of HDAC11 ablates downstream targets and abrogates self-renewal of stem-like cells

**Figure 4:**
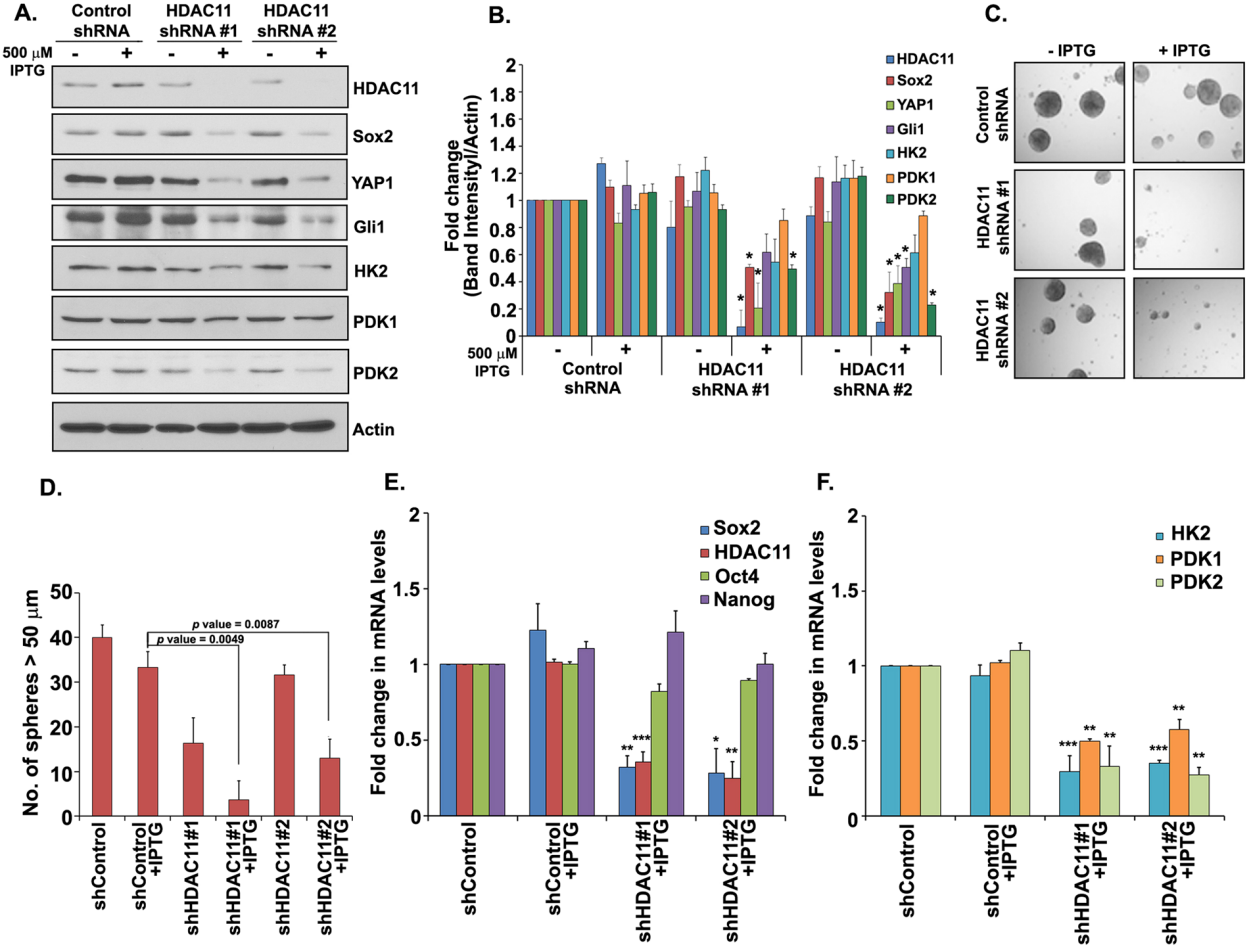
HDAC11 silencing decreases expression of downstream targets and abrogates self-renewal of CSCs. (**A**) Depletion of HDAC11 using two IPTG inducible shRNA shows a significant decrease in Sox2, YAP1 and Gli1 protein expression as compared to IPTG treated control shRNA. The glycolysis pathway targets HK2 and PDK2 also show reduced protein expression in the absence of HDAC11. No change is observed in PDK1 expression; the full images of the western blots are provided as Supplementary Fig. 4. (**B**) Quantitation of the western blot band intensities using ImageJ analysis. (**C**) Sphere formation assay with SP cells from shHDAC11 H1650 cells treated with IPTG show abrogation of self-renewal ability in the absence of HDAC11. (**D**) Quantitation of sphere formation assay reveal that IPTG treated SP cells from two different clones of HDAC11 shRNA containing H1650 form fewer spheres as compared to IPTG untreated or control shRNA containing cells. (**E**,**F**) Real time PCR analysis of H1650 cells with two different HDAC11 shRNA show a decrease in mRNA expression of *Sox2*, *HK2*, *PDK1*, *PDK2* with IPTG treatment. No significant change was observed in *Oct4* and *Nanog* expression. The depletion of HDAC11 was confirmed using RT-PCR.

Since the earlier results indicated that HDAC11 could induce Sox2-luc promoter activity, we attempted to investigate the role of HDAC11 in expression of Sox2 and its potential downstream targets. H1650 cells were stably transduced with two different IPTG-inducible HDAC11 shRNA. Treatment of the cells with 500 μM IPTG for 5–7 days efficiently depleted HDAC11; there was a concomitant reduction in Sox2 protein levels, as seen by western blotting (Fig. 4A). We also observed a decrease in the expression of YAP1 and Gli1 proteins upon depletion of HDAC11 (Fig. 4A). This was further confirmed by quantitation of the band intensities using ImageJ analysis software (Fig. 4B). Since it has been suggested that Sox2 might affect energy metabolism in stem-like cells, we examined if depleting HDAC11 might affect any metabolic genes that were potentially downstream of Sox2^31,32^. A promoter analysis of glycolysis pathway genes like *hexokinase (HK2)*, *Pyruvate dehydrogenase kinase1 (PDK1)* and *2 (PDK2)* presented several Sox2 transcription factor binding sites (*data not shown)*, suggesting that they are potential transcriptional targets of Sox2. We assessed the expression of these metabolic genes in IPTG treated HDAC11 depleted cells; HK2 and PDK2 showed a significant decrease in their protein expression with the depletion of HDAC11 (Fig. 4A,B). At the same time, there was minimal change in expression of PDK1 at the protein level (Fig. 4A,B).

Since HDAC11 expression was elevated in the SP cells, we examined if depletion of HDAC11 affected the self-renewal of these cells. SP cells isolated from H1650 cell line stably expressing HDAC11 shRNA were treated with 500 μM IPTG in stem cell selective media for 10 days. It was found that the depletion of HDAC11 reduced the self-renewal of CSCs markedly; IPTG treatment had no effect on SP cells isolated from H1650 cell line stably expressing a non-targeting, control shRNA (Fig. 4C). Quantitation of the spheres revealed that the reduction in sphere formation was significant (Fig. 4D).

Given the above result, we examined if the stem cell transcription factors as well as Sox2 target genes were affected by stable depletion of HDAC11. RT-PCR experiments showed a significant decrease in *Sox2* mRNA expression (Fig. 4E) with no similar changes in *Oct4* and *Nanog* expression in HDAC11 knock down cells upon treatment with IPTG (Fig. 4E). Similar reduction was also observed in the levels of *HK2*, *PDK1* and *PDK2* mRNAs, compared to IPTG treated shControl cells (Fig. 4F). These results suggested that HDAC11 function was necessary for the self-renewal of SP cells and this was probably mediated through the expression of Sox2 and its targets.

### Selective HDAC11 inhibitors reduced stem-like properties of CSCs

**Figure 5:**
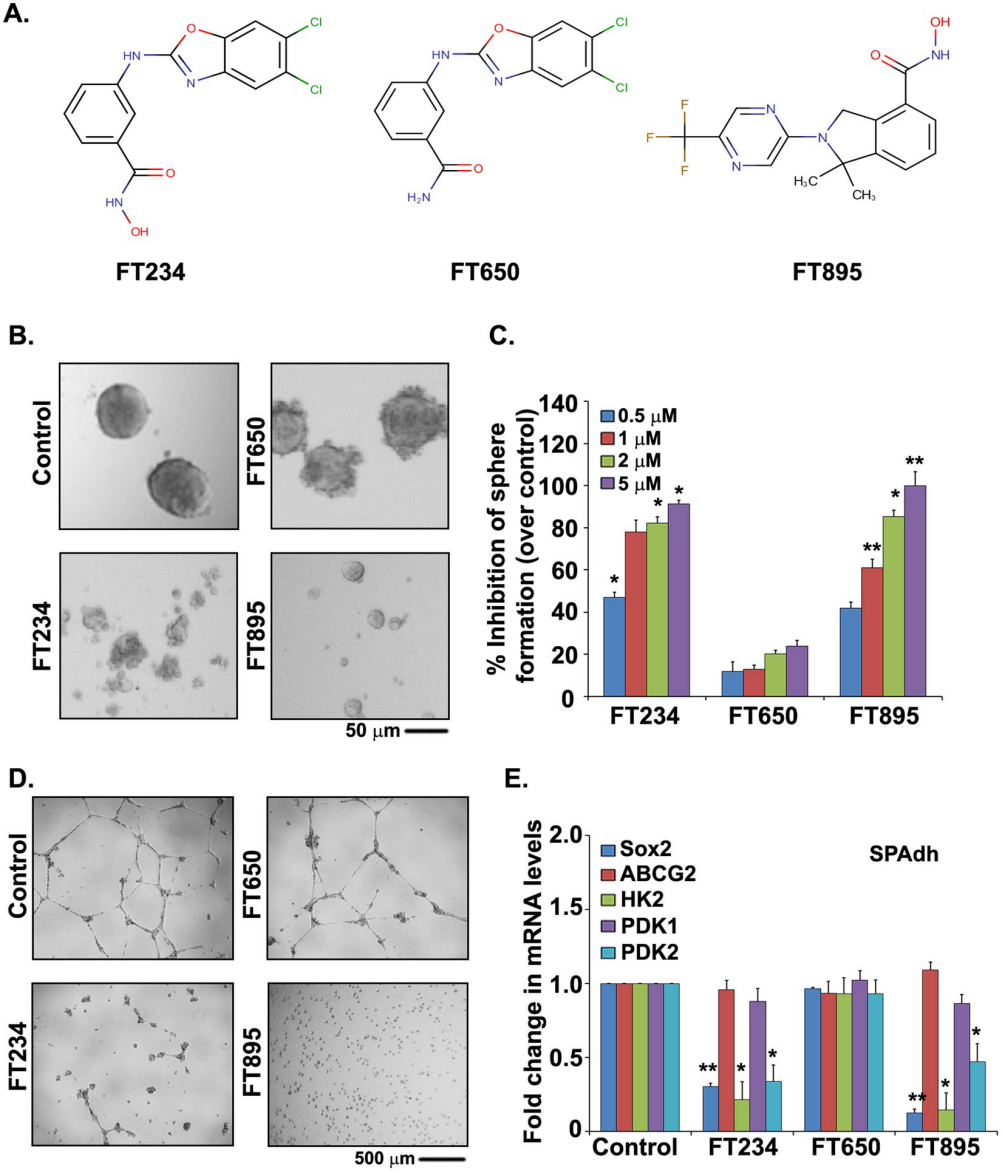
Selective HDAC11 inhibitors ablates stem-like properties of cancer stem cells. (**A**) Structures of the three HDAC11 inhibitors used in the study. (**B**) Sphere formation assay with SP from H1650 cells in the presence of 2 μM concentration of HDAC11 inhibitors FT234 and FT895 show a significant reduction in self-renewal abilities of these cells. FT650 did not have an effect on the self-renewal. (**C**) Quantitation of the sphere assay confirmed the reduction in the number of sphere formed by H1650 SP cells in the presence of various concentrations of HDAC11 inhibitors FT234 and FT895. The inhibitor FT650 did not affect the self-renewal ability (**D**) H1650 SP cells treated with 5 μM concentration of HDAC11 inhibitors FT234 and FT895 show abrogation of angiogenic tubule-like structure formation when grown on Matrigel in stem cell medium. FT650 had minimal effect on vascular mimicry of H1650 SP cells. (**E**) RT-PCR analysis of 2.5 μM HDAC11 inhibitor FT234 and FT895 treated SPAdh reveal decrease in *Sox2* and metabolic targets *HK2* and *PDK2* mRNA expression as compared to DMSO control treatment. FT650 had no effect as seen before.

To further investigate inhibition of HDAC11, we utilized highly selective and potent inhibitors of HDAC11 those were developed by FORMA Therapeutics^33,34^. We focused on two of these inhibitors FT234 and FT895 (Fig. 5A); the structurally related FT650 was used as an inactive control.

Treatment of the stem-like SP cells with 2μM of the selective HDAC11 inhibitors FT234 and FT895 led to a significant reduction in self-renewal as compared to the DMSO treated control (Fig. 5B). The structurally similar but inactive compound FT650 had minimal effect on self-renewal (Fig. 5B). Quantitation of the number of spheres formed in the presence of various concentrations of FT234, FT895 and FT650 revealed a dose dependent inhibition upon treatment with FT234 as well as FT895 (Fig. 5C). No such effect was observed with FT650 (Fig. 5C).

It has been shown that CSCs can undergo vascular mimicry where they are able to form vasculogenic networks on matrigel^35,36^. Our earlier studies had shown that side population isolated from H1650 cells can undergo vascular mimicry efficiently^17,18^. There was a significant reduction in the formation of vascular networks by SP cells when treated with 5 μM of FT234 or FT895, as compared to DMSO treatment (Control) (Fig. 5D). A treatment of SP cells with 5 μM of FT650 did not have an effect on the vascular mimicry (Fig. 5D). Thus, it appears that these highly selective inhibitors of HDAC11 could significantly reduce self-renewal of stem-like cells and inhibit their trans-differentiation into vascular cells.

To investigate the essential genes and regulatory pathways influenced by HDAC11 inhibitors in stem-like SP cells, we attempted to generate adherent cultures of the SP cells as described in our earlier publications^17,37^. Such SP-adherent (SPAdh) cells maintained SP phenotype in 80% of the cells up to 8 days in culture^17^. When these SPAdh cells were treated with HDAC11 selective inhibitors FT234 and FT895, there was a significant decrease in the mRNA of *Sox2* as well as its target genes like *HK2* and *PDK2*, as compared to SPAdh cells treated with either DMSO control or the inactive FT650 compound (Fig. 5E). These experiments suggest that inhibition of HDAC11 can lead to a downregulation of Sox2 as well as its metabolic targets in the stem-like cells, abrogating self-renewal as well as the vascular mimicry of SP cells.

### HDAC11 inhibitors prevent tumor cell migration and anchorage independent growth

**Figure 6:**
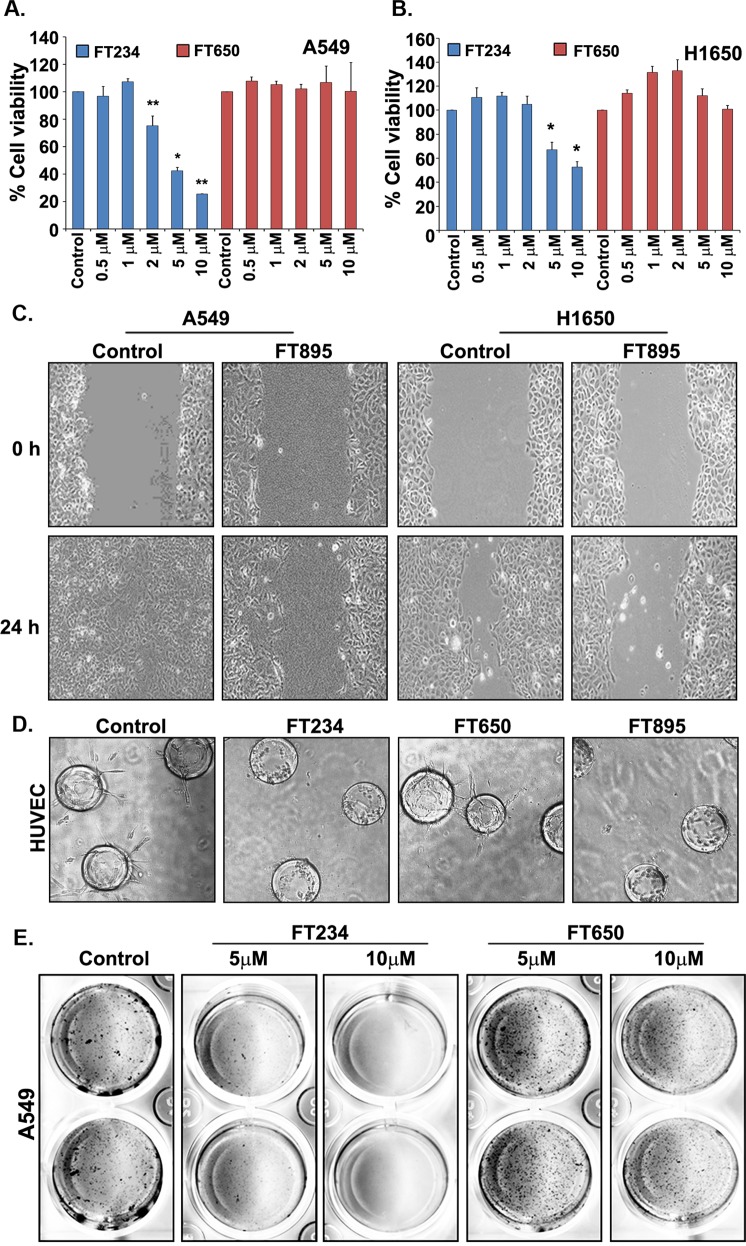
HDAC11 inhibitors prevent tumor cell growth, viability and migration. (**A**,**B**) Cell viability assays performed on A549 (**A**) and H1650 (B) cells with various concentrations of FT234 show a significant decrease in viability of both the cell lines at 5 and 10 μM concentrations. The negative control FT650 had no such effect. (**C**) Treatment of A549 and H1650 cells with 10 μM FT895 for 24 h abrogates the ability of these cells to migrate in a wound healing assay as compared to the DMSO control treated cells. (**D**) HDAC11 inhibitors FT234 and FT895 at 10 μM concentration prevent angiogenic tubular extensions in HUVEC cells in a FIBA assay. The negative control FT650 had minimal effect on the tubule growth. (**E**) The anchorage independent growth of A549 cells assessed in a soft agar colony formation assay in the presence of FT234 and FT650 show a significant decrease in the number of colonies formed in FT234 treatment as compared to DMSO Control treatment. The number of colonies in FT650 treatment as negative control was comparable to DMSO treatment.

We next examined the effect of HDAC11 inhibitors on overall cell viability by performing MTT assays. Our results show that 5–10 μM of FT234 compound inhibited the growth and viability by 60–80% in both A549 (Fig. 6A) as well as H1650 cells (Fig. 6B) as compared to the DMSO control treated cells. The treatment with negative control FT650 had no effect on the viability of both the cells (Fig. 6A,B). To further understand the efficiency of the inhibitors, we estimated the IC_50_ values for cell viability in the presence of the inhibitors in multiple cell lines namely, A549, H1650, AALE (immortalized tracheobronchial cells) and primary lung CAFs (Supplementary Fig. 2). Table 1 showed the IC_50_ values for cell viability. The overall IC_50_ value in the primary cells (AALE and lung CAFs) was higher as compared to the cancer cells (Table 1). Also, the FT650 didn’t affect the viability of the cells tested (Supplementary Fig. 3 and Table 1). The IC_50_ values for the cancer cell lines A549 and H1650 ranged from 4.663–6.594 µM whereas in AALE the values were 10.74 µM (FT894) and 15.11 µM (FT234). The IC_50_ values for primary lung CAFs were 21.34 µM (FT894) and 99.82 µM (FT234) (Supplementary Fig. 3 and Table 1). It should be mentioned that off-target effects are a possibility at these high doses tested in the normal cells. It is likely that the more mesenchymal features of the CAFs contribute to the reduced sensitivity to the HDAC11 inhibitors.

**Table 1:** IC_50_ value for cell viability of cells treated with HDAC11 inhibitors.

Cell Lines	Cell Type	HDAC11 Inhibitor	IC_50_ Values for MTT Assay
A549	Lung adenocarcinoma	FT234	6.594 µM
		FT650	NA
		FT895	4.663 µM
H1650	Lung adenocarcinoma	FT234	4.917 µM
		FT650	NA
		FT895	4.777 µM
Lung cancer associated fibroblasts (CAFs)	Fibroblasts	FT234	99.82 µM
		FT650	NA
		FT895	21.34 µM
AALE	Immortalized tracheobronchial epithelial	FT234	15.11 µM
		FT650	NA
		FT895	10.74 µM

Cell migration and invasion are essential for tumor metastasis^38,39^. To investigate the effect of the HDAC11 inhibitors on cell migration, a wound healing assay was performed on plastic in the presence of the HDAC11 inhibitors. It was found that cells treated with 10 μM FT895 for 24 h migrated at a slower rate as compared to the DMSO control treated A549 and H1650 cells (Fig. 6C). It is also known that migration is an essential feature in the process of neoangiogenesis and vessel development by the endothelial cells during tumor growth^40^. We performed a 3D fibrin gel bead assay (FIBA assay) to assess the effect of the HDAC11 inhibitors on angiogenic tubule formation. Treatment with the HDAC11 inhibitors FT234 and FT895 prevented the growth of tubular networks formed by the primary HUVEC cells, as compared to cells treated with DMSO control and the inactive compound FT650 (Fig. 6D). RT-PCR experiments showed that the VEGF receptors, Flt-1 and KDR were suppressed by the HDAC11 inhibitors (data not shown), suggesting a possible mechanism by which angiogenic tubule formation is inhibited by these agents.

Additional experiments were conducted to examine the effect of HDAC11 inhibitors on anchorage independent growth, as this feature strongly correlates with increased tumorigenicity of cancer cells^41^. Ability of A549 cells to form colonies in soft agar was measured using standard protocols, in the presence of FT234 and FT650. Treatment with 5μM or 10μM the of the compounds for 21 days showed that FT234 could eliminate the anchorage independent growth of A549 cells at both 5 and 10 μM concentrations whereas the inactive control FT650 had no effect on the growth, as compared to the control cells (Fig. 6E). These results strongly suggest that inhibiting HDAC11 using these specific inhibitors might be beneficial in combating cancer, due to their ability to reduce self-renewal, vascular mimicry, migration and adherence-independent growth.

### HDAC11 inhibitors reduce Sox2 protein and mRNA expression in cells

**Figure 7:**
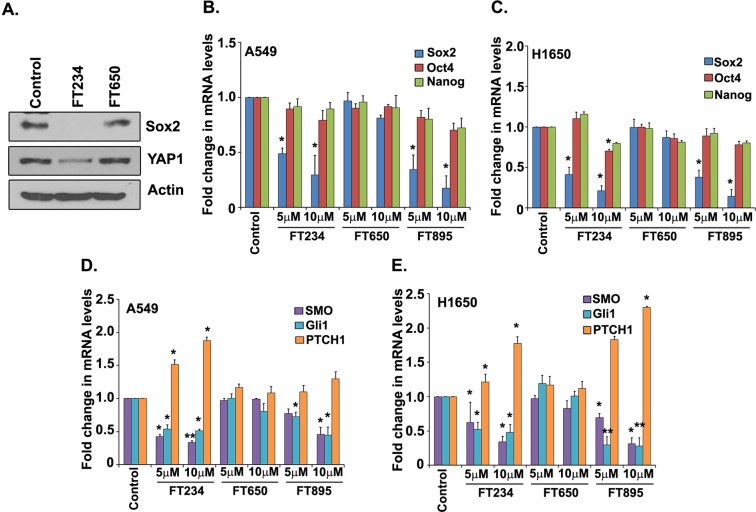
HDAC11 inhibitors reduces Sox2 expression and its regulatory pathways in cells. (**A**) Western blot analysis with lysates of cells treated with 5 μM of FT234 for 48 h reveal a significant decrease in Sox2 protein expression. There was no change in the expression of Sox2 with FT650 treatment. The full images of the western blots are provided as Supplementary Fig. 5. (**B**,**C**) RT-PCR analysis of A549 (**B**) and H1650 (**C**) treated with 5 and 10 μM for 72 h with FT234 and FT895 show a significant decrease in *Sox2* mRNA expression. There was minimal or no change in *Oct4* and *Nanog* expression. The negative control FT650 also showed no change in the mRNA expression of the ES transcription factors. (**D**,**E**) RT-PCR analysis of FT234 and FT895 treated A549 (**D**) and H1650 (**E**) reduce the expression of *Gli1* and *SMO* with the treatment. Simultaneously, there was an increase in *PTCH1* expression with FT234 and FT895 treatment. As observed earlier, there was no change with the FT650 treatment.

Since depletion of HDAC11 selectively suppressed Sox2 expression, experiments were conducted to examine if the HDAC11 inhibitors could affect the expression of Sox2 and its target genes in a similar fashion. A western blot analysis showed that treatment of H1650 cells with 5 μM of FT234 for 72 h decreased Sox2 and YAP1 protein expression as compared to the control cells (Fig. 7A). The FT650 compound had no effect on the expression of either Sox2 or YAP1 (Fig. 7A).

We next assessed if these specific HDAC11 inhibitors could affect the mRNA levels of the ES transcription factors. The treatment of A549 and H1650 cells with FT234 as well as FT895 reduced the mRNA expression of *Sox2* transcription factor (Fig. 7B-C); FT650 didn’t have any effect. Also, there was no change in the expression of *Oct4* and *Nanog* mRNA in A549 and H1650 cells treated with FT234 or FT895 (Fig. 7B-C). This observation was similar to that of depleting HDAC11, suggesting that these novel inhibitors of HDAC11 could exert a similar effect as depleting the protein.

Given the effect of HDAC11 depletion of Gli1 protein, we next examined the effect of FT234 and FT895 on the hedgehog pathway components. Expression of *Gli1* mRNA was significantly reduced upon treatment with FT234 and FT895 in both A549 and H1650 cells (Fig. 6D,E). A similar decrease was observed in the levels of *Smoothened (Smo)* mRNA as well, in both A549 and H1650 cells (Fig. 7D,E). Interestingly, a corresponding increase was observed in the expression of *Patched1 (PTCH1)* mRNA in A549 and H1650 cells upon treatment with these inhibitors (Fig. 7D,E). Patched1 is known to suppress hedgehog signaling by inhibiting Smoothened^19,42^ and hence it appears that the HDAC11 inhibitors are downregulating the oncogenic components of the hedgehog pathway, while elevating the levels of the growth suppressive *PTCH1*.

### HDAC11 inhibitors reduce the viability of EGFR TKI resistant cells

**Figure 8:**
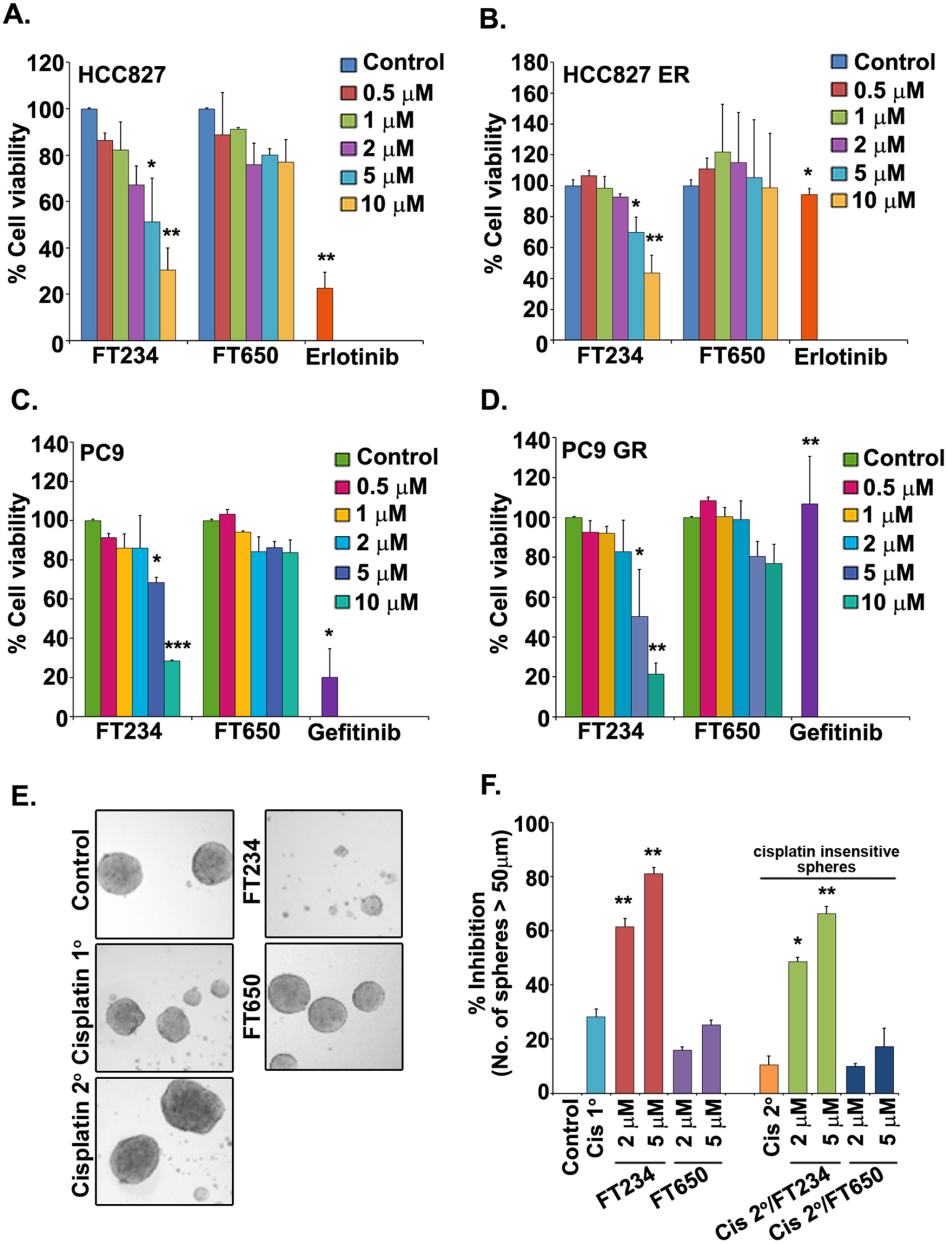
HDAC11 inhibitors reduce the viability of chemo-resistant cancer cells as well as chemo-insensitive CSCs. (**A**,**B**) Cell viability assays with HCC827 parental (**A**) and HCC827 ER (**B**) cells treated with various concentrations of FT234 show decrease in viability in both the parental and resistant cells at 10 μM concentration as compared to DMSO control treatment. FT650 was a negative control and Erlotinib was used as a positive control. (**C**,**D**) Cell viability assays with PC9 parental (**C**) and PC9 GR (**D**) cells treated with 10 μM of FT234 reveal a decrease in viability as compared to DMSO control. Gefitinib was a positive control and FT650 was used as a negative control here. (**E**) Sphere formation assay with FT234 treatment in Cisplatin-insensitive (Cisplatin 2°) cells prevent their self-renewal at 5 μM concentration. (**F**) Quantitation of the sphere formation assay with cisplatin-insensitive (cisplatin 2°) cells show ablation of number of spheres formed by the cisplatin-insensitive (cisplatin 2°) in the presence of FT234 inhibitor. Such significant decrease was not observed with FT650 treatment.

Development of drug resistance is widespread in NSCLC patients, and resistance can occur against common chemotherapeutic drugs as well as targeted therapies^3,7,43,44^. EGFR mutations are prevalent in NSCLC and patients harboring these mutations respond robustly to EGFR TKI inhibitors like erlotinib or gefitinib^4,45,46^. However, they eventually develop resistance to these drugs, resulting in the resurgence of highly metastatic, drug resistant tumors^45^. It is widely believed that CSCs contribute to the development of drug resistance and cancer recurrence^10,13^; since HDAC11 inhibitors could inhibit the self-renewal of CSCs, reduced cell viability and adherence-independent growth of NSCLC cells, we next examined if these inhibitors could eliminate cells those were resistant to EGFR inhibitors. We performed MTT assays on parental HCC827 and erlotinib resistant HCC827 (ER) cells. As can be seen in the Fig. 8A,B, 10 μM FT234 could significantly reduce the viability of HCC827 ER cells (58%), comparable to the parental HCC827 cells (67%). The 2 μM erlotinib treatment eliminated the parental HCC827 cells (Fig. 8A) but not the erlotinib resistant HCC827 ER cells (Fig. 8B). We also conducted similar experiments on viability of parental PC9 cells and gefitinib-resistant PC9 (GR) cells. Here too, 10 μM FT234 significantly reduced the viability of both the parental PC9 cells by (72%) (Fig. 8C) and the PC9 GR cells (80%) (Fig. 8D). 2 μM gefitinib treatment reduced the viability of the parental cells, but not the PC9 GR cells (Fig. 8C,D). These results clearly indicated that HDAC11 inhibitor FT234  could efficiently reduce the survival of the TKI sensitive as well as the resistant cells.

Platinum compounds are widely used as chemotherapeutic agents to treat advanced NSCLC and cisplatin is especially efficacious in this regard^47^. However, cisplatin treatment results in acquired resistance to this drug by promoting multi-drug resistance in CSCs^48^. Given our observations that HDAC11 inhibitors could eliminate the SP cells that were generally drug resistant, experiments were conducted to assess if HDAC11 inhibitors could eliminate stem-like cells that were insensitive to cisplatin-mediated cytotoxicity. The cisplatin-insensitive cells were generated by treating the SP cells with 5 μM of cisplatin (Supplementary Fig. 4; Materials and Methods). As can be seen from the Fig. 7E,F, treatment with 2 μM and 5 μM of FT234 could inhibit the self-renewal of SP cells that were insensitive to prior cisplatin treatment. The cisplatin insensitive spheres showed comparable inhibition of self-renewal (70%) of the SP cells as the FT234 treatment alone (80%) (Fig. 8F). The FT650 compound had no effect on the self-renewal of SP cells with or without cisplatin treatment. Our earlier studies had shown that Sox2 levels are upregulated in drug resistant cells, and it is likely that inhibition of HDAC11 reduces Sox2 expression, conferring sensitivity to the drugs. These results suggest that inhibition of HDAC11 is a useful avenue to eliminate stem-like cells that are insensitive or resistant to standard chemotherapeutic agents.

### HDAC11 inhibitors selectively prevent growth of cancer cells in presence of CAFs

**Figure 9:**
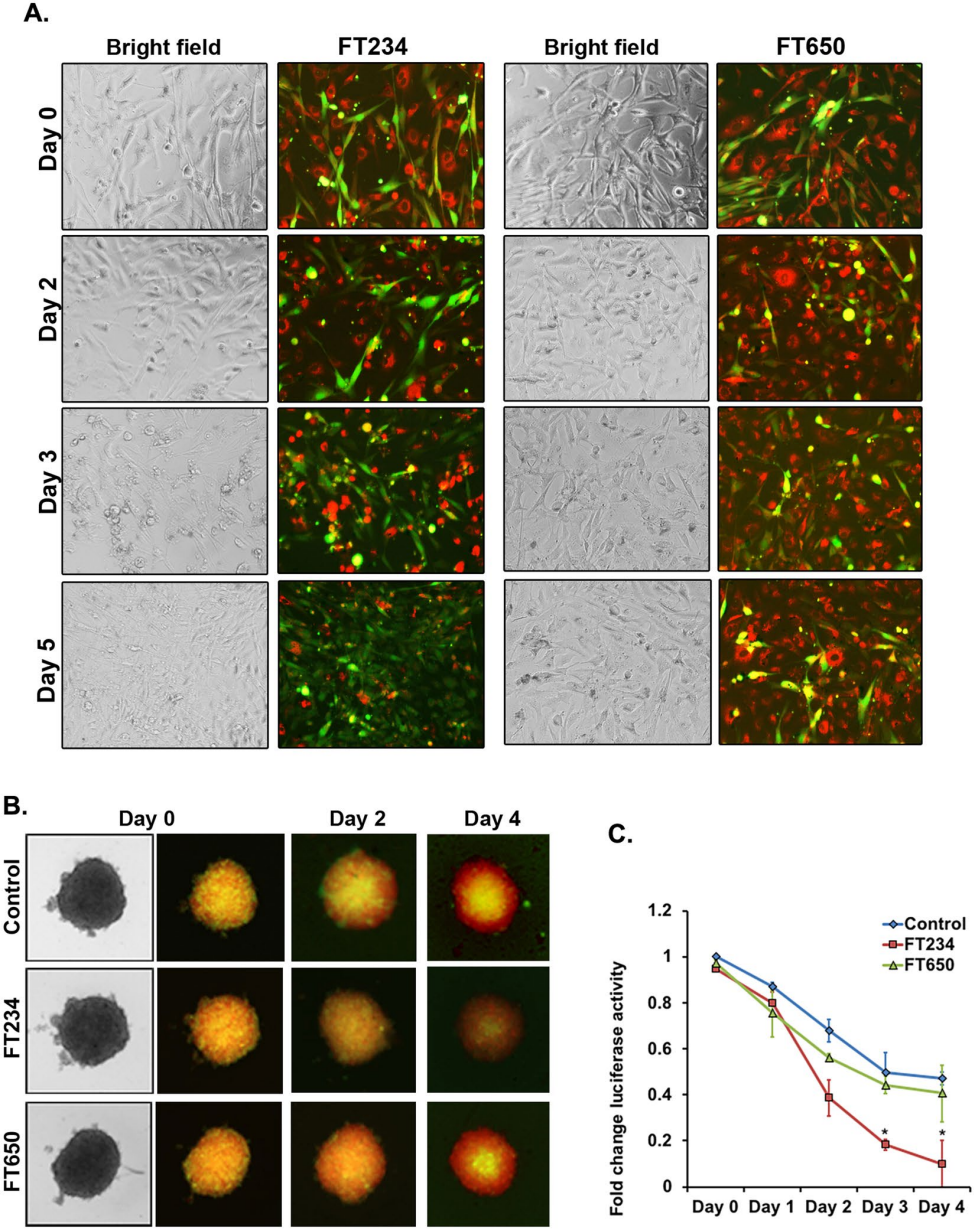
HDAC11 inhibitors prevent growth of cancer cells even in presence of CAFs. (**A**) Treatment of 2D coculture of A549 (red) cells with primary lung CAFs (Green) with 10 μM of FT234 show that the HDAC11 inhibitor selectively reduces the growth of A549 cells as compared to the Control co-cultures in 48 h. Such an effect was not observed in the negative control FT650 treatment. (**B**) 3D coculture assay with labeled A549-luc cells (red) and primary lung CAFs (Green) in non-adherent conditions show a decrease in the growth of A549-luc cells in the presence of 10 μM FT234 in 4 days as compared to control co-cultures. The FT650 did not affect the growth of A549 or CAF cells. (**C**) Quantitation of the luciferase activity as a measure of the viability and growth of the A549-luc cells in the presence of the HDAC11 inhibitors reveal that FT234 reduces the luciferase activity of these cells as compared to the DMSO control (*p* = 0.0164) or the structurally similar negative control FT650 (*p* = 0.0261).

Components of the tumor microenvironment like cancer associated fibroblasts, endothelial cells and immune cells can modulate the growth and progression of tumors^49^. Cancer associated fibroblasts (CAFs) are known to render drug resistance in various tumor types including NSCLC, mainly through the secretion of growth factors and other survival signals^49^. We therefore examined the efficacy of the HDAC11 inhibitors on reducing the viability of cancer cells in the presence of primary human lung CAFs. Towards this purpose, we labelled A549 or H1650 lung cancer cell lines with CellTracker red dye and the CAFs with CellTracker green dye and co-cultured them on tissue culture plates in the presence or absence of HDAC11 inhibitors. The results showed that the 10 μM of FT234 inhibitor could selectively prevent the growth of the A549 or H1650 cells (labeled red) by day 3 even in the presence of lung CAFs (labeled green), as seen by the reduction in the red cells and the enrichment of green CAFs (Fig. 9A, Supplementary Fig. 4A). The FT650 compound didn’t inhibit the growth of A549 or H1650 cells, and there appeared to be more red cells compared to green CAFs after three days in co-culture (Fig. 9A, Supplementary Fig. 4A). This experiment suggests that FT234 may eliminate cancer cells selectively even when untransformed CAFs are present.

Given that tumors grow in three dimensions, we examined if HDAC11 inhibitors could eliminate cancer cells selectively in 3D cultures as well. The 3D co-culture assays were carried out in both A549 and H1650 cells. The A549 cells used were stably transfected with a luciferase gene (A549-luc) to enable their quantitation. Both A549-luc and H1650 were labeled with CellTracker red dye and primary lung CAFs were labeled with CellTracker green dye and co-cultured in 1:1 ratio in the presence or absence of the HDAC11 inhibitors in non-adherent condition. The results showed that the 10 μM of FT234 could inhibit the growth and viability of the A549-luc as well as H1650 cells even in the presence of the CAFs in the 3D co-cultures (Fig. 9B, Supplementary Fig. 4B). FT650 did not inhibit the viability of either the cancer cells or the lung CAFs. The inhibition of the growth and viability by HDAC11 inhibitors was monitored in A549-luc cells by measuring luciferase activity using luciferin as a substrate. As seen in Fig. 9C, FT234 treatment significantly reduced luciferase activity in A549 cells as compared to the FT650 or the DMSO control treatment, indicating that HDAC11 inhibitors can efficiently prevent the growth of A549 cells even when CAFs are present. The results confirm the efficacy of the HDAC11 inhibitors against drug insensitive CSCs that may arise during the chemotherapeutic treatments, indicating that HDAC11 inhibitors might be potentially useful in treating NSCLC.

## Discussion

The current treatment strategies for NSCLC have several disadvantages including lower efficacy of treatment and tumor resurgence due to drug resistance to chemotherapeutic agents^7,45,46^. Immunotherapy has been successful only to smaller subset of lung cancer patients^4,50^. Hence it is imperative to identify novel therapeutic strategies to combat this disease. This is also due to the fact that KRAS mutant cancer lacks effective therapeutic interventions. Though there are potent inhibitors to treat EGFR or ALK mutant lung cancer patients, they eventually develop resistance to these inhibitors^6,43^. It has been suggested that drugs that can eliminate CSCs would be effective as anti-cancer agents and can potentially overcome drug resistance^45,51^.

In recent years, there has been a significant increase in understanding the role of histone modifications and their contribution to gene regulation during normal cellular homeostasis as well as in diseases like cancer^52,53^. Histone acetylation plays a crucial role in chromatin remodeling and affects gene transcription. Histone acetylation involves the dynamic balance between two group of enzymes, histone acetyl transferases (HATs) and HDACs^20^. Numerous studies have shown that in several types of cancers, overexpression of HDACs correlate with decrease in disease-free survival and could predict poor patient prognosis^23^. Based on these observations, HDACs have been successfully targeted for cancer therapy; agents like Vorinostat, Romidepsin and Panobinostat, have been approved as a therapeutic for various hematologic malignancies^22,54^. Unfortunately, the HDAC inhibitors developed so far have not been highly efficacious against lung cancer^52^. HDAC inhibitors are known to regulate the expression of various genes involved in inhibiting cancer development and progression at the transcriptional level, by altering the acetylation status of histone cores in the promoter region^21^. It has also been suggested that HDACs have a role in maintenance of CSCs^28^. Thus, given their ability to affect the transcription of vital genes and alter the viability of CSCs, HDAC inhibitors are promising candidates for cancer therapy.

HDAC11 is the sole member of the class IV HDAC family and has homology to both class I and II HDACs^53^. The results presented here reveal higher expression of HDAC11 in the CSCs from NSCLC and depletion of HDAC11 decreased the self-renewal abilities of the CSCs. Our results show the role of HDAC11 in regulating Sox2 expression in lung cancer cells, with a limited effect on *Oct4* and *Nanog* expression. Our earlier studies had shown that Sox2 is not only higher in metastatic lung tissue but is also indispensable for the survival of the CSCs^17–19^. HDAC11 also showed cooperativity with previously reported pathways regulating *Sox2* gene expression in lung cancer^18,19^. Our finding that the novel HDAC11 inhibitors described here can eliminate the self-renewal of stem-like cells and can target even drug resistant lung cancer cells suggest that these novel agents and their derivatives would be of potentially useful in combating NSCLC.

An added benefit of the HDAC11 inhibitors described here is their ability to target vascular mimicry in addition to self-renewal of stem-like cells. While, the HDAC11 inhibitors might be affecting the self-renewal by reducing Sox2 expression, the effect on the vascular mimicry may be due to reduction of VEGF receptors Flt1 and KDR upon treatment with these HDAC11 inhibitors. Thus, the ability of these drugs to target cell proliferation, adherence-independent growth, self-renewal and vascular mimicry contribute to their value as anti-cancer agents. The observation that HDAC11 inhibitors can downregulate vital metabolic genes indicated that this could be a mechanism by which these inhibitors reduce cell viability, as seen in the MTT assays. While Sox2 might not be directly involved in the survival of differentiated cancer cells, its downstream targets like HK2 and PDK2 might be contributing in viability of the cells through their key roles in metabolic processes. Recently, HDAC11 has been also been shown to have role in metabolic homeostasis^55–57^. The ability of the tested compounds to downregulate the metabolic genes through HDAC11 and Sox2 might be a feature that will enable these inhibitors to eliminate stem-like and non-stem-like cancer cells simultaneously. Thus, it is likely that HDAC11 inhibitors, either alone or in combination, would be effective as anti-cancer agents, by suppressing multiple targets in the cells.

In a similar vein, our results also show an inhibitory effect of the HDAC11 inhibitors on hedgehog pathway and the expression of Gli1 transcription factor. The effect of HDAC11 inhibitors on the hedgehog pathway indicates a potential for using these inhibitors alone or in combination with hedgehog pathway inhibitors for treatment of NSCLC. The finding that HDAC11 physically interacts with Gli1 adds a new dimension to this possibility; indeed, it remains to be seen if this interaction between Gli1 and HDAC11 affects the expression of additional genes other than Sox2. Additionally, in this study we have assessed the growth of TKI resistant cells in the presence of the HDAC11 inhibitors. It is known that lung cancer patients develop resistance to TKI therapy due to secondary mutations in the EGFR gene or activation of alternative signaling pathways. The HCC827 erlotinib resistant cells and the PC9 gefitinib resistant cells used in this study developed c-Met amplification and EGFR T790M gatekeeper mutation respectively as mechanisms of resistance against TKIs^58,59^. The HDAC11 inhibitors could prevent the growth of these TKI resistant cells effectively. The HDAC11 inhibitors also inhibited the growth of H1975 cells which harbor the T790M gatekeeper mutation in the *EGFR* gene *(data not shown)*. The effectiveness of the HDAC11 inhibitors against the TKI resistant lung cancer cell lines shows a potential of such inhibitors to be used in patients with TKI resistance. Also, many chemotherapeutic treatment regimens lead to drug insensitive CSCs. The HDAC11 inhibitors could prevent the self-renewal of cisplatin-insensitive CSCs showing their activity on resistant as well as drug insensitive population of cancer cells. While the above result clearly demonstrate the potential utility of these agents to combat lung adenocarcinomas, it is not fully clear why the HDAC11 inhibitor had a lower efficacy on CAFs as well as AALE cells. It is likely that the mesenchymal features of the CAFs reduce their efficacy; similarly, the AALE cells are immortalized, which might contribute to their differential response. There was also a disparity between the efficacy of the inhibitor and the levels of HDAC11 in certain cells, probably due to differing mutational landscape present in them. Additional studies will reveal the molecular basis for these differences; at the same time, it is clear that the inhibitor phenocopies the depletion of HDAC11, suggesting that their effects are mostly through targeting HDAC11 itself, modulating its downstream targets.

It is becoming increasingly evident that the tumor microenvironment plays an essential role in cancer progression^60^. CAFs are crucial part of the tumor microenvironment and many reports suggests that CAFs mediate resistance to therapy in case of solid tumors like NSCLC; further, they secrete chemokines, cytokines and growth factors, stimulating tumor growth and metastasis^49^. Our findings that HDAC11 inhibitors could efficiently eliminate cancer cells even in the presence of CAFs suggest that the inhibitors can overcome the survival benefits provided by the CAFs. This observation also raises the possibility that these inhibitors have selectivity to cancer cells might be of immense value for cancer therapeutics.

The HDAC11 inhibitors presented here are novel and highly selective. They have low IC_50_ values against HDAC11 activity^33^. At the same time, their ADME properties need to be further optimized for conducting *in vivo* experiments. The *in vivo* toxicity profile of these compounds also remains to be established. In conclusion, this study presents a mechanistic basis for the role of HDAC11 in lung adenocarcinoma and suggests that once the parameters for *in vivo* studies and efficacy are met, they would be of immense potential in combating NSCLC.

## Materials and Methods

### Cells and reagents

Human NSCLC cell lines H1650, HCC827, H358, H460 and PC9 were purchased from ATCC and maintained in RPMI 1640 (Gibco, Life Technologies) supplemented with 10% fetal bovine serum (Seradigm, VWR International). A549 cells were purchased from ATCC and were maintained in Ham’s F12K medium (Cell Gro, Corning) supplemented with 10% fetal bovine serum. The AALE cells (tracheobronchial epithelial cells) were maintained in bronchial epithelial growth medium (BEGM) containing growth supplement (Lonza). The primary lung CAFs were obtained from Neuromics (#CAF07A) and were cultured in low serum VitroPlus III medium (Fisher Scientific). The drug resistant HCC827 ER cell line was maintained in 1 µM erlotinib (#S1023, Selleck Chemicals) in culture media and PC9 GR was maintained in 1 µM gefitinib (#G4408, LC laboratories) in the media^59^. All the cultures were maintained at 5% CO_2_ at 37 °C.

### Antibodies

The antibodies Sox2 (#3579), Oct4 (# 2750), Hexokinase 2 (#2106), Acetylated Histone H3 (# 9671 S) and Gli1 (#2643) were from Cell Signaling Technologies. The pyruvate dehydrogenase kinase 1 (PDK1) (#ab47987) and 2 (PDK2) (#ab68164) antibodies were from Abcam; YAP1 antibody was from Abnova (H00010413-M01), HDAC11 (NBP2-16789) was from Novus Biologicals; mouse monoclonal antibody (A1978) to actin was purchased from Sigma-Aldrich.

### Tissue microarray and immunohistochemistry

Human NSCLC tissue array (NBP2-30222, Novus Biologicals) had 59 cores including normal lung (9 cores), NSCLCs (40 cores) and metastatic NSCLCs (10 cores). The immunohistochemical staining for HDAC11 (NBP2- 16789) was carried out according to previously published protocols^18,61^. The second tissue microarray (MC1801, US BioMax) with 26 NSCLC cores and 10 normal lung tissue cores was also stained for HDAC11. Both the slides were scanned on Aperio automatic scanning system from Applied Imaging and were scored by a pathologist. For HDAC11 and Sox2 staining, two human tissue arrays (NBP2-30222, Novus Biologicals) were used. The staining was carried out simultaneously to keep the conditions similar.

The IHC scoring was based on the Allred score system^62^. Briefly, the semi-quantitative score was derived by considering both cellularity and intensity of HDAC11 expression in the tissues. The staining intensity ranged between 0 to 3, 0 being negative, 1 being weak, 2 being moderate, and 3 as strong staining. The cellularity was scored as follows: a score of 5 indicating that greater than 67% of tissue was positive, score of 4 indicating positivity in 34–66% of tissue, score of 3 indicating positivity in 11–33% of tissue, score of 2 is equal to 1–10% of tissue and a score of 1 is less than 1% of tissue. We used the summation of both the scores as the total score for HDAC11 staining for each TMA core. For Sox2 and HDAC11 correlation analysis, the Pearson correlation coefficient (*r*) value was found to be 0.5041. To calculate the statistical significance of the results obtained unpaired two tailed Student’s *t-* test was use for the TMA analyses.

### KM Plots and survival analysis

The Kaplan Meier survival analysis was performed using the KM Plotter dataset^24^ (http://kmplot.com/analysis/index.php?p=service&cancer=lung). For every gene from this dataset^24^, each percentile of expression between the lower and upper quartiles were computed and the best performing threshold was used as the final cutoff in a univariable Cox regression analysis.

### Lysate preparation and western blot analysis

The cells were washed with ice-cold PBS, scraped and centrifuged at 800 *g* and lysed using M2 lysis buffer (20 mM Tris-HCl pH 6.0, 0.5% NP-40, 250 mM NaCl, 3 mM EGTA and 3 mM EDTA) containing protease inhibitors^61,63^. The protein content was estimated using Bradford assay (Bio-Rad). 50 μg protein from the cell lysates was separated using SDS-PAGE and transferred to nitrocellulose membrane (Bio-Rad Transblot Semi-dry). The membranes were blocked with 5% non-fat dry milk in PBS with 0.1% Tween-20 and incubated with appropriate primary antibodies. 1:3000 diluted HRP-conjugated secondary antibodies (Thermofisher Scientific) were used and signals were detected using ECL (Thermofisher Scientific). To quantitate the band intensities of the western blot bands, ImageJ analysis was performed (ImageJ Software).

For the HDAC11 inhibitor treatment, the indicated inhibitors were added to the cells at 5 μM concentration with control treatment being an equal volume of DMSO. The cells were harvested after 72 hours of treatment and further processed for lysate preparation and western blotting. The inhibitor treatments followed by western analysis was performed twice.

### Isolation of side-population cells and generation of SP Adherent (SPAdh) cells

To isolate side population cells, asynchronously growing cells were harvested using Accutase reagent (Innovative cell technologies, Inc.), washed once with DPBS and re-suspended in DMEM:F12K medium (Gibco, Life Technologies) with 2% FBS at 1 ×10^6^ cells/ml density. Cells were then incubated with 4 μg/ml of Hoechst 33342 dye (Life Technologies) for 90 min at 37 °C in the presence or absence of 1 μM Fumitremorgin C (Sigma Aldrich). The side population (SP) cells were sorted using FACS Vantage (BD FACSDiVa) cell sorter as described in previous publications^17,18^. Data analyses were done using the FlowJo software (Tree Star). The isolated cells were further used for various experiments.

To generate the SP Adherent (SPAdh) cells, sorted SP cells were grown on Poly-D-Lysine and Laminin coated plates (Sigma Aldrich) in stem cell selective media [DMEM:F12K (1:1) supplemented with N2 supplement (1×) (Invitrogen), 10 ng/ml EGF and 10 ng/ml bFGF (Sigma Aldrich)]^17^. The HDAC11 inhibitor treatments on SPAdh cells were carried out at 2.5 μM concentrations for 96 hours and were further analyzed by RT-PCRs.

### Sphere formation assay for self-renewal

The sorted cells (SP) were plated in ultralow attachment 96-well plate (Corning) at the density of 1000 cells/100 μl/well in stem cell selective medium at 37 °C for 10 days^17–19^. The spheres were observed and imaged using Evos FL microscope system and images were acquired with EVOS software (Life Technologies Inc., USA). The number of spheres greater than or equal to 50 μm was counted.

To study the effect of the HDAC11 inhibitors on the self-renewal ability of SP cells, appropriate concentrations were added to the respective wells on Day 0 and the size and number of the spheres were analyzed on Day 10. The sphere formation assays were performed twice in triplicates for each treatment in the assay.

To generate the cisplatin insensitive CSCs, the SP cells were grown in the presence of 5 μM of Cisplatin in stem cell selective media under low adherence for 7–10 days. The cisplatin insensitive spheres were collected and dissociated into single cell suspension using 0.05% trypsin EDTA solution (Corning). They were re-plated at a density of 1000 cells in 100 μl of stem-cell selective medium in the presence or absence of HDAC11 inhibitors or Cisplatin and allowed to further grow in low adherence conditions for 7–10 days (Supplementary Fig. 4). The number of spheres greater than or equal to 50 μm was counted as mentioned above.

### siRNA transfections

The siRNAs for HDAC1 (sc-29343) HDAC6 (sc-35544) and HDAC11 (sc-106896) were purchased from Santa Cruz Biotechnologies. The siRNAs were transfected at a concentration of 100 pmoles each into the cells using Oligofectamine reagent (Invitrogen) as per manufacturer’s protocol. A non-targeting siRNA (Ambion AM4635) was used as a control for all the transfection experiments. The cells were harvested after 48 h post transfection for different assays. All the siRNA experiments were performed thrice.

### RNA Isolation and qRT-PCR analysis

Total RNA was isolated from the cells by RNeasy Miniprep kit from Qiagen following the manufacturer’s protocol. 1 µg of RNA was converted into cDNA using iScript cDNA synthesis kit (Bio-Rad). Levels of mRNA were analyzed using quantitative reverse transcription-PCR (qRT-PCR) that was performed using Bio-Rad CFX96 Real time system. Data was normalized using GAPDH as an internal control and fold change was calculated by 2^−ΔΔCt^ method. In case of HDAC11 inhibitor treatments, the cells were treated with the indicated concentrations of the inhibitors for 72 h following which they were harvested for RNA isolation as mentioned above. The primers used in this study are as follows:

HDAC1 FP -5′ GACGGACCGACTGACGGTAG 3′,

HDAC1 RP -5′ ATTTCCAACATCCCCGTCGT 3′,

HDAC6 FP -5′ TGGCGGAGTGGAAGAACC 3′,

HDAC6 RP -5′ GTTCTGCCTACTTCTTCGCTG 3′,

HDAC11 FP -5′ CGGCCAGCTTTGGGAGG 3′,

HDAC11 RP -5′ GGCCCATGAAGGTGATGTTG 3′,

Sox2 FP 5′-GGGAAATGGGAGGGGTGCAAAAGA-3′,

Sox2 RP 5′-TTGCGTGAGTGTGGATGGGATTGG-3′,

Oct4 FP 5′-ACATCAAAGCTCTGCAGAAAGAACT-3′,

Oct4 RP 5′-CTG AAT ACC TTC CCAAAT AGA ACC C-3′,

Nanog FP 5′-AGAAGGCCTCAGCACCTA-3′,

Nanog RP 5′-GGCCTGATTGTTCCAGGATT-3′,

GAPDH FP 5′-GGTGGTCTCCTCTGACTTCAACA-3′,

GAPDH RP 5′-GTTGCTGTAGCCAAATTCGTTGT-3′

HK2 FP 5′-GGACTTCCTCGAGTACATGGG-3′,

HK2 RP 5′-TGAGGAGGATGCTCTCGTCCA-3′,

PDK1 FP 5′-TTATGCTGTATGGCCTGCAAGA-3′,

PDK1 RP 5′-CAATCACACCCTGGGCCATT-3′,

PDK2 FP 5′-CGGGGACCACAACCAAAGTC-3′,

PDK2 RP 5′-AGTGCTCTATGTACTTGGGCG-3′,

ABCG2 FP 5′-CACAAGGAAACACCAATGGCT-3′,

ABCG2 RP 5′-ACAGCTCCTTCAGTAAATGCCTTC-3′,

FLT1 FP 5′-CTGGCTCCTATTAACCCTCCTTA-3′,

FLT1 RP 5′-ATTTGCCCAGTTTAAGTCTCTCC-3′,

KDR FP 5′-TGGAAGTGAGTGAAAGAGACACA-3′,

KDR RP 5′-TACTGGTAGGAATCCACAGGAGA-3′,

MMP2 FP 5′-CCGCCTTTAACTGGAGCAAAAA-3′,

MMP2 RP 5′-GATGAGCTTGGGGAAGCCAG-3′,

MMP9 FP 5′-CGGAGCACGGAGACGGGTATC-3′,

MMP9 RP 5′-GGGCAGAGTAGGAGCGGCCCT-3′,

MMP14 FP 5′-GGATACCCAATGCCCATTGGCCAG-3′,

MMP14 RP 5′-CCATTGGGCATCCAGAAGAGAGC-3′,

MMP15 FP 5′-CAGCCCAGCCGCCATATGTC-3′,

MMP15 RP 5′-CTTTCACTCGTACCCCGAAC-3′,

YAP1 FP 5′-CCCAAGACGGCCAACGTGCC-3′,

YAP1 RP 5′-ACTGGCCTGTCGGGAGTGGG-3′,

Gli1 FP 5′-CCCAATCACAAGTCAGGTTCCT-3′,

Gli1 RP 5′-CCTATGTGAAGCCCTATTTGCC-3′,

PTCH1 FP 5′-CGGCCGGCTATGGGGA-3′,

PTCH1 RP 5′-ACCAAGAACTTGCCGCAG-3′,

SMO FP 5′-CGCTACAACGTGTGCCTGG-3′,

SMO RP 5′-CGGAGGCCCGACCAGAG-3′,

### Transfections and luciferase assays

*Sox2* proximal promoter-luciferase construct was kindly provided by Dr. Angel G. Martin (Inbiomed, Spain)^64^. Flag tagged HDAC11 expression vector was a kind gift of Dr. Ed Seto^65,66^. Gli1 (TCH1003, Transomic) was subcloned into pcDNA3 vector as mentioned previously^19^. pcDNA4/HisMaxB-YAP1 was a gift from Marius Sudol (Addgene plasmid # 18978)^67^.

For the luciferase reporter assays, cells were transiently transfected using FugeneHD (Promega) according to the manufacturer’s protocol. Luciferase assays were carried out after 48 h post-transfection using the dual-luciferase assay system (Promega) as per manufacturer’s instructions. Luciferase activity was measured using a luminometer (Glomax MultiJR detection system). For each experiment, the relative luciferase activity was measured as the ratio of the *Firefly* luciferase to *Renilla* luciferase and the fold changes were calculated compared to the control luciferase vector alone from at least three independent experiments.

### ChIP assays

ChIP assays were conducted on asynchronous cells as described previously using indicated antibodies^18,19,63,68^. The interactions at the promoter were analyzed using qRT-PCR analysis. The sequences of the ChIP PCR primers are as follows:

Gli1 ChIP FP 5′-TCCTGATTCCAGTTTGCCTC-3′,

Gli1 ChIP RP 5′-GGGAGAGGAGGAGGGGAG-3′,

Myc ChIP FP 5′ CCCCAACAAATGCAATGGGAG 3′,

Myc ChIP RP 5′ CAGAGCGTGGGATGTTAGTG 3′

For the ChIP assays on side population (SP) cells from H1650, the SP cells were sorted first using flow cytometry as described earlier and then grown in stem cell media for 10 days. The spheres were then used for ChIP analysis.

### Generation of stable HDAC11 depletion using inducible HDAC11 shRNA

The two IPTG inducible shRNAs clones 199149 and 330863 against HDAC11 (mission shRNA #SHCLNG-NM_024827) were kind gifts from Dr. Kenneth Wright (Moffitt Cancer Center, Tampa, FL) and were originally purchased from Sigma Aldrich. The lentiviral particles with the shRNAs were produced by packaging them with psPax2 and pMD2.G vectors. H1650 cells were transduced with the viral particles and were selected for the positive clones with 2 μg/ml of puromycin after 72 h post-transduction.

For the knockdown of HDAC11, the puromycin selected H1650 cells were treated with 500 μM of IPTG for 5–7 days by adding IPTG to the medium every 48 h. The knock down of HDAC11 was confirmed by RT-PCRs and western blot analysis.

To carry out the self-renewal assay with HDAC11 knock down cells, the puromycin selected H1650 cells were stained with Hoechst 33342 as mentioned above and Hoechst negative SP cells were sorted by flow cytometry. The sorted cells were further grown in the stem cell selective media for 10 days in the presence or absence of 500 μM IPTG. IPTG was added on Day1, 4 and Day 7 to the stem cell medium.

### Tubule formation assay for vascular mimicry

The Hoechst negative sorted stem-like SP cells were allowed to differentiate on Matrigel (BD Biosciences) to form tubule-like structures^17,18^. For this, 100 µl of thawed growth factor reduced Matrigel (CB40230A, Corning) was layered in the wells of 96-well tissue culture plates followed by incubation for 30 min at 37 °C to allow polymerization. The sorted cells at a density of 12000 cells in 100 µl of stem cell medium were layered on the polymerized Matrigel and incubated for 6–8 at 37 °C. Tubule formed representing vascular mimicry was assessed in bright field using EVOS FL microscope system and images were acquired with EVOS software (Life Technologies Inc., USA). For the inhibitor treatment, 5 μM HDAC11 inhibitors were added to the medium while seeding the SP cells on Matrigel.

### Cell viability assay

Cell viability was measured using MTT (Thiazolyl Blue Tetrazolium Bromide) after 96 hours of indicated treatment with HDAC11 inhibitors. Briefly, the cells were plated in 96-well plates at a density of 3000 cells/well in triplicates. After treatments, they were incubated with the 0.5 mg/mL MTT solution at 37 °C for 30 min to 1 h. The formazan crystals were dissolved in 150 μl DMSO and absorbance was measured at 590 nm using a plate reader. For the IC_50_ value estimation, a concentration range of 20 µM to 0.039 µM was used for the compound treatment in all the cells lines namely A549, H1650, AALE and Lung CAFs and a 10-point curve was plotted using GraphPad Prism software to calculate the IC_50_ values.

### Wound healing assay

A549 and H1650 cells were grown in 6-well plates (BD Biosciences) to 90–95% confluency. A wound was created using a sterile 2 µl pipette tip in each well. The cells were washed and further incubated with media containing 10μM of indicated HDAC11 inhibitors at 37 °C. After 24 h, images were taken using EVOS FL microscope system and EVOS software (Life Technologies Inc).

### Fibrin gel bead assay

To evaluate angiogenic tubule formation in 3D, a fibrin gel bead assay was performed^69^. Briefly, HUVECs were coated and cultured on cytodex beads (Sigma-Aldrich) in endothelial growth medium. The endothelial cell-coated beads were then embedded into fibrin matrix which was formed using fibrinogen, aprotinin and thrombin. A layer of 12,000 aortic smooth muscle cells were seeded on the fibrin matrix to support the growth of endothelial cells. For the HDAC11 inhibitor treatment, initial sprouting was monitored for 24 h and 10 μM of selective HDAC11 inhibitors were added once the initial sprouts were formed. The tubule formation was evaluated after 24 hours using an EVOS FL microscope system and images were acquired EVOS software.

### Soft agar colony formation assay

The soft agar colony formation assays were carried out in triplicates in 12-well cell culture plates (Corning). For this assay, first layer of 0.6% agar is allowed to solidify at room temperature after which 0.3% agar mixed with 5000 cells (A549) and indicated HDAC11 inhibitors per well was layered on top. The mentioned concentrations of HDAC11 inhibitors were also added to each well in 10% FBS containing media. The media with the fresh inhibitors were replenished every 72 h and colonies were allowed to grow over a period of 21 days. The colonies were visualized using 1 mg/ml MTT solution^63,70^.

### and 3D co-culture assays of lung cancer cells with primary lung CAFs

For the co-culture assays, lung cancer cells (A549 or H1650) were labeled with CellTracker red (C34552) and the primary lung CAFs were labeled with CellTracker green (C2925) stains as per manufacturer’s protocol (Life Technologies). Briefly, the 1×10^6^ cells/ml were incubated with each of the 10μM CellTracker stains at 37 °C for 45–60 mins. Once the cells are labeled, the cells were washed and then cancer cells (A549 or H1650) and primary lung CAFs were mixed in 1:1 ratio. In the 2D co-culture assays, a total of labeled 5000 cells/well were seeded in a 96-well plate and allowed to grow for 8–12 hours. The cells were then incubated further in the presence or absence of indicated HDAC11 inhibitors and the viability of the cells was evaluated every 24 h using the EVOS FL microscope system. The images were acquired EVOS software every 24 h.

For the 3D co-culture assay, A549 cells stably expressing a luciferase gene (A549-luc) were generated. For this the cells were transfected with pcDNA3.1-luciferase construct and were selected with neomycin to stably express the firefly luciferase gene^68^. The cells were labeled as mentioned above. 500 labeled cell mix (1:1) in 100 μl media per well were incubated in round bottom low adherence 96-well plates in the presence or absence of the HDAC11 inhibitors. The growth of the cells in the presence of inhibitors was monitored every 24 hours using the EVOS FL microscope system and the images were acquired with EVOS software. To measure the luciferase activity of A549-luc cells, we used the One Glo EX luciferase assay system (E8110, Promega) as per manufacturer’s instructions.

### Statistical analysis

All data have been statistically analyzed using Microsoft Office Excel 2010 (Microsoft Corporation, Redmond, WA). The data presented here is with ±standard deviation (SD) values unless otherwise stated. The statistical comparisons between the groups were carried out by unpaired two tailed Student’s *t*-test or one-way ANOVA to calculate the *p* value for statistical significance. The ChIP qPCR was analyzed using two-way ANOVA test. *p < 0.05, **p < 0.01 and ***p < 0.001.

## Supplementary information

Supplementary Information.
