# Supplementary material for: Novel HDAC11 inhibitors suppress lung adenocarcinoma stem cell self-renewal and overcome drug resistance by suppressing Sox2
Source: Sci Rep. 2020 Mar 13;10:4722. doi: 10.1038/s41598-020-61295-6 (PMC7069992; doi:10.1038/s41598-020-61295-6)
Supplement: Supplementary file 1 — Supplementary Information. [file 41598_2020_61295_MOESM1_ESM.docx]

**Novel HDAC11 inhibitors suppress lung adenocarcinoma stem cell self-renewal and overcome drug resistance by suppressing Sox2**

**­**

**Namrata Bora-Singhal, Durairaj Mohan Kumar, Biswarup Saha, Christelle Colin, Jennifer Y. Lee, Matthew W. Martin, Xiaozhang Zheng, Domenico Coppola and Srikumar Chellappan**

**SUPPLEMENTARY INFORMATION**

**Supplementary Figures**

**
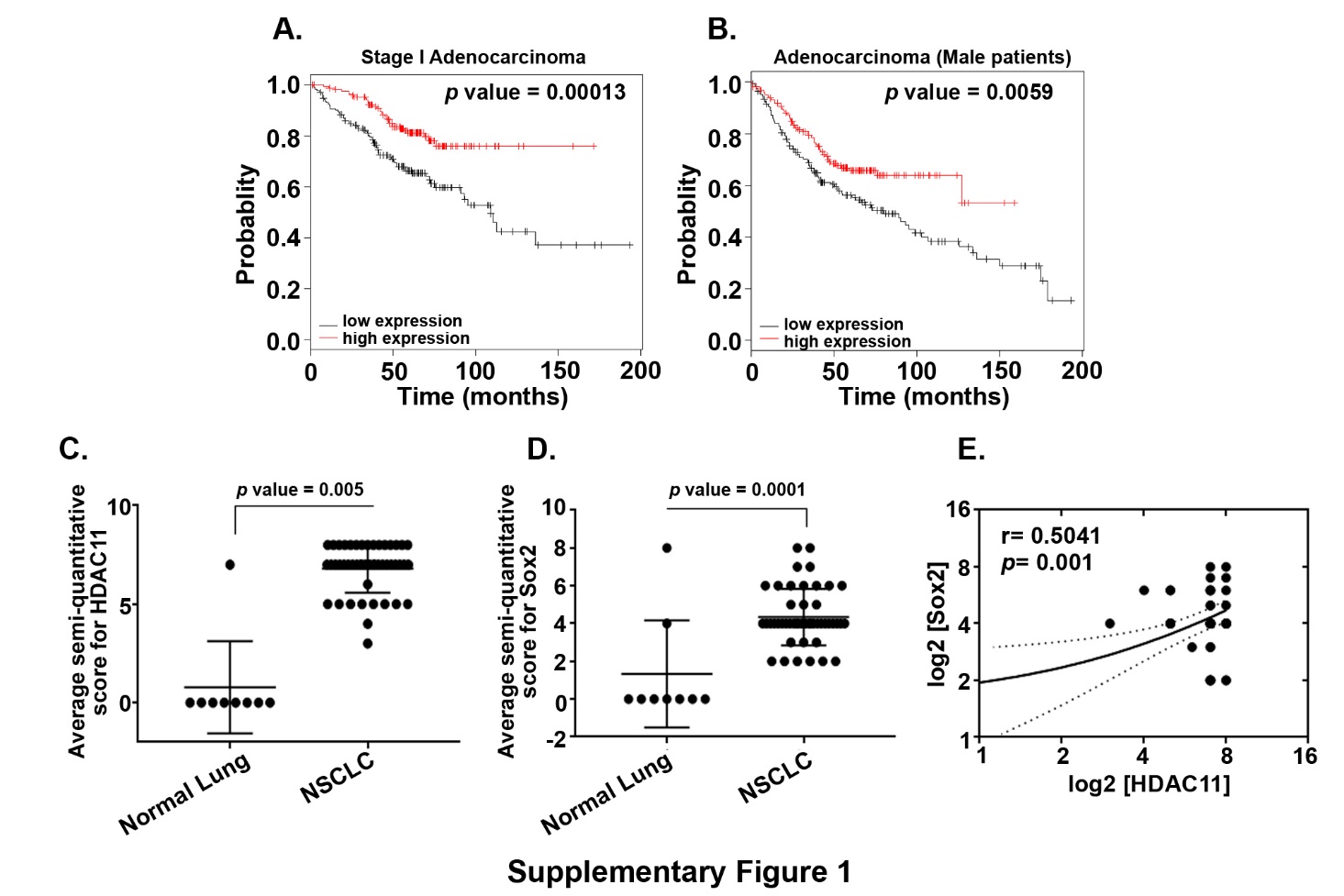
**

**Supplementary Figure 1. Expression of HDAC11 in NSCLC patients predicts poor survival.**

**(A-B)** Kaplan Meier multivariate survival analysis showed poor survival for early stage (Stage 1) lung adenocarcinoma patients (A). The poor survival rates were also observed in male lung adenocarcinoma patients with higher expression of HDAC11 (B). **(C-E)** Higher expression of HDAC11 (C) and Sox2 (E) was observed in NSCLC patients. The Pearson correlation coefficient analysis showed a moderately positive correlation coefficient (r) value (E) in the NSCLC patients tumor tissue.

**
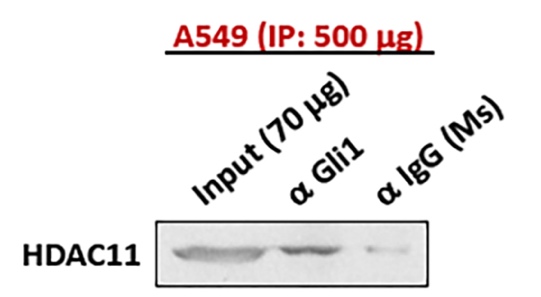
**

**Supplementary Figure 2.** An immunoprecipitation-western blot experiment on A549 cells showing the association of Gli1 with HDAC11. 500μg of lysates were immunoprecipitated with an anti-Gli antibody or a control IgG and the immunoprecipitate probed for the presence of HDAC11 by western blotting. 70μg of the lysate was loaded as the input lane on the gel.

**
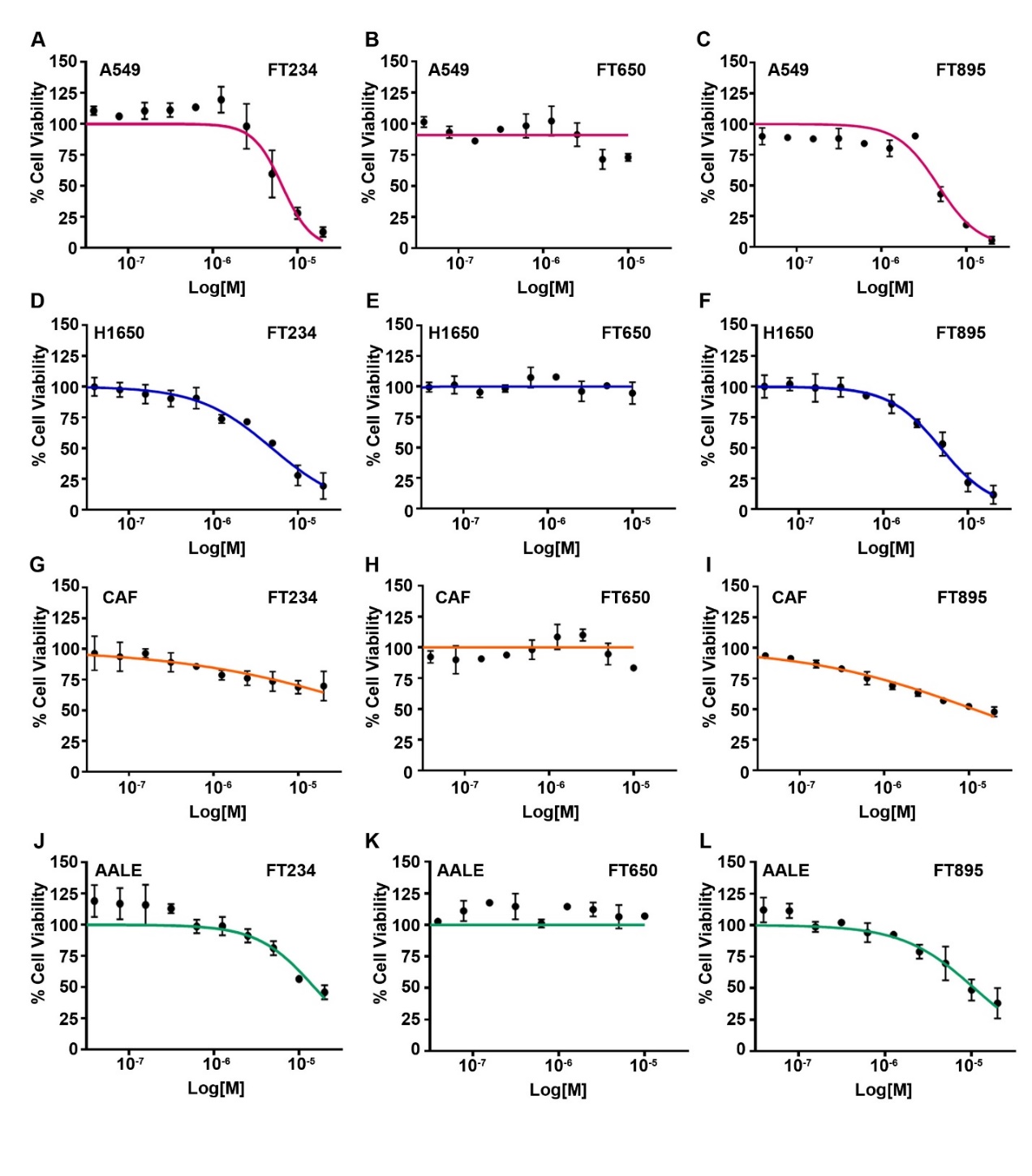
**

**Supplementary Figure 3.**

**(A-L)** 10-point dose curves for cell viability to estimate IC_50_ values for the cell lines namely A549 (A-C), H1650 (D-F), lung CAF (G-I) and AALE (J-L).

**
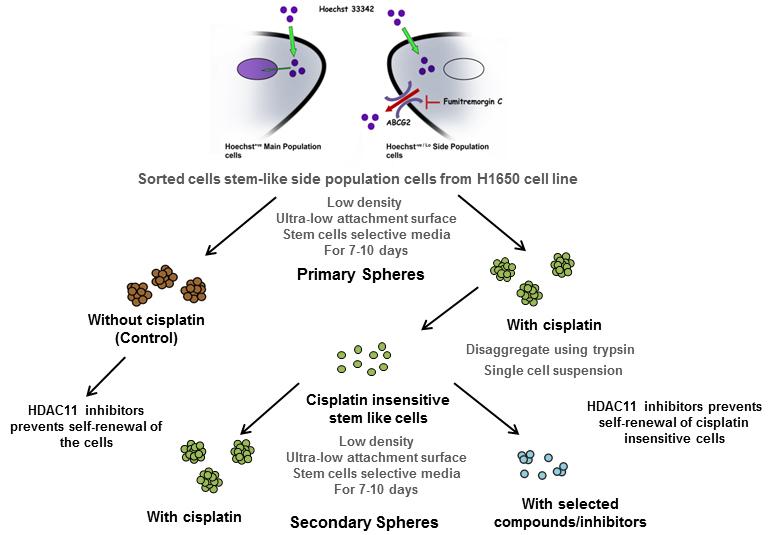
**

**Supplementary Figure 4. Schematic to show the generation of cisplatin – insensitive stem-like cells.**

Heochst negative SP from H1650 cells were sorted by flow cytometry and plated in the presence or absence of 5 μM Cisplatin for 7-10 days in ultra-low attachment surface in stem-cell media. The spheres of SP cells that grew in presence of cisplatin are referred as cisplatin insensitive stem-like cells (Primary spheres). These cells were dissociated and re-plated in stem-cell media in ultra-low adherence for another 7-10 days in the presence of HDAC11 inhibitors to assess the effect of the inhibitors on cisplatin-insensitive cells. These cells were also grown in the presence of 5 μM Cisplatin separately as positive control.

**
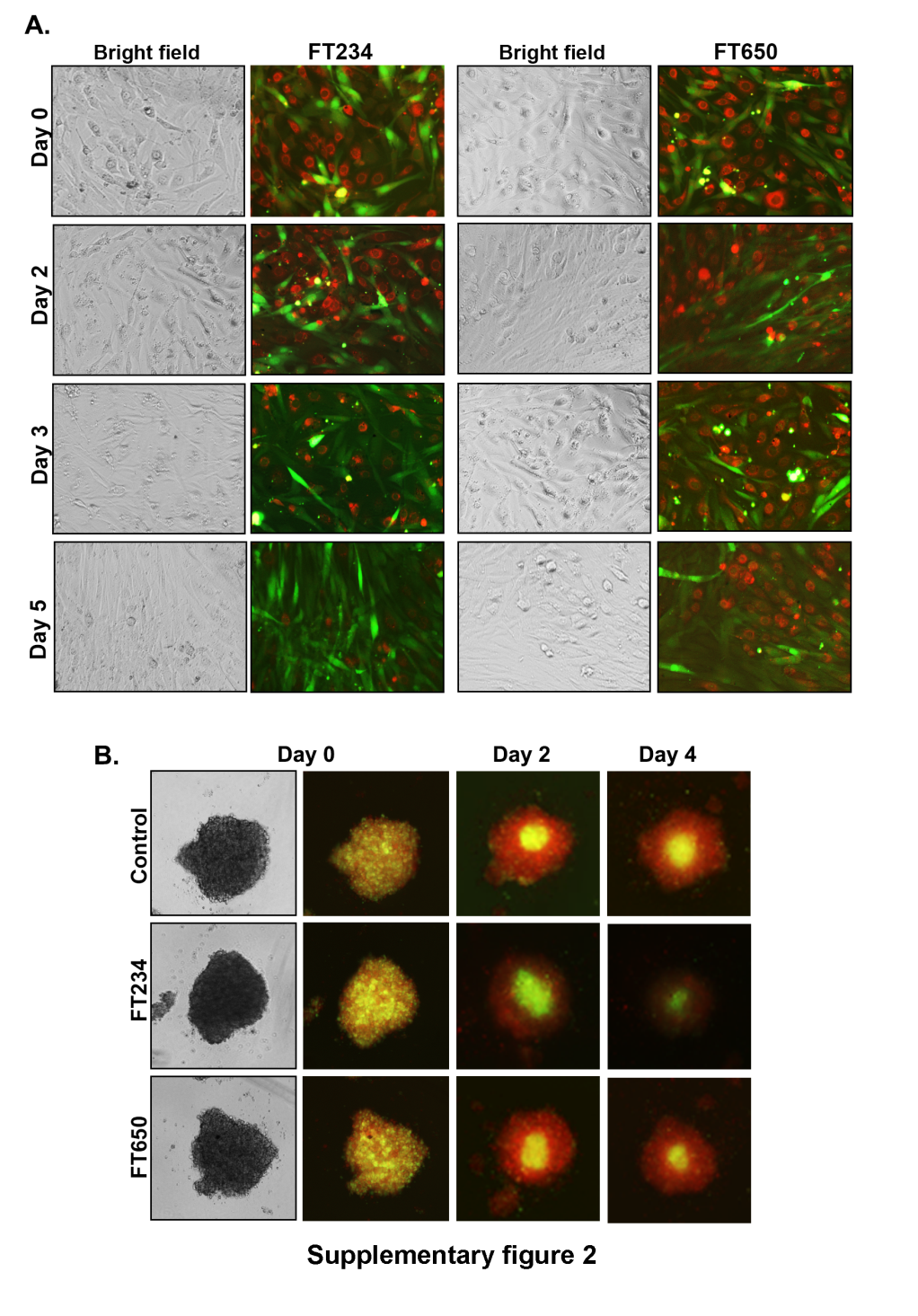
**

**Supplementary Figure 5. HDAC11 inhibitors reduce the growth of H1650 cells in the presence of lung CAFs.**

1. The treatment of HDTK010 compound show a decrease in growth and viability of the H1650 cells (red) in 72 h even in the presence of the primary lung CAFs (green) in 2D co-culture assay. The negative control HDTK069 did not affect the growth of the H1650 cells. (B) The HDAC11 inhibitor HDTK010 prevents the growth of H1650 cells (red) in the presence of CAFs (green) in 3D non-adherent cultures by day 4. Such an effect was not observed in HDTK069 treatment.

**Western Blots – Full scans**


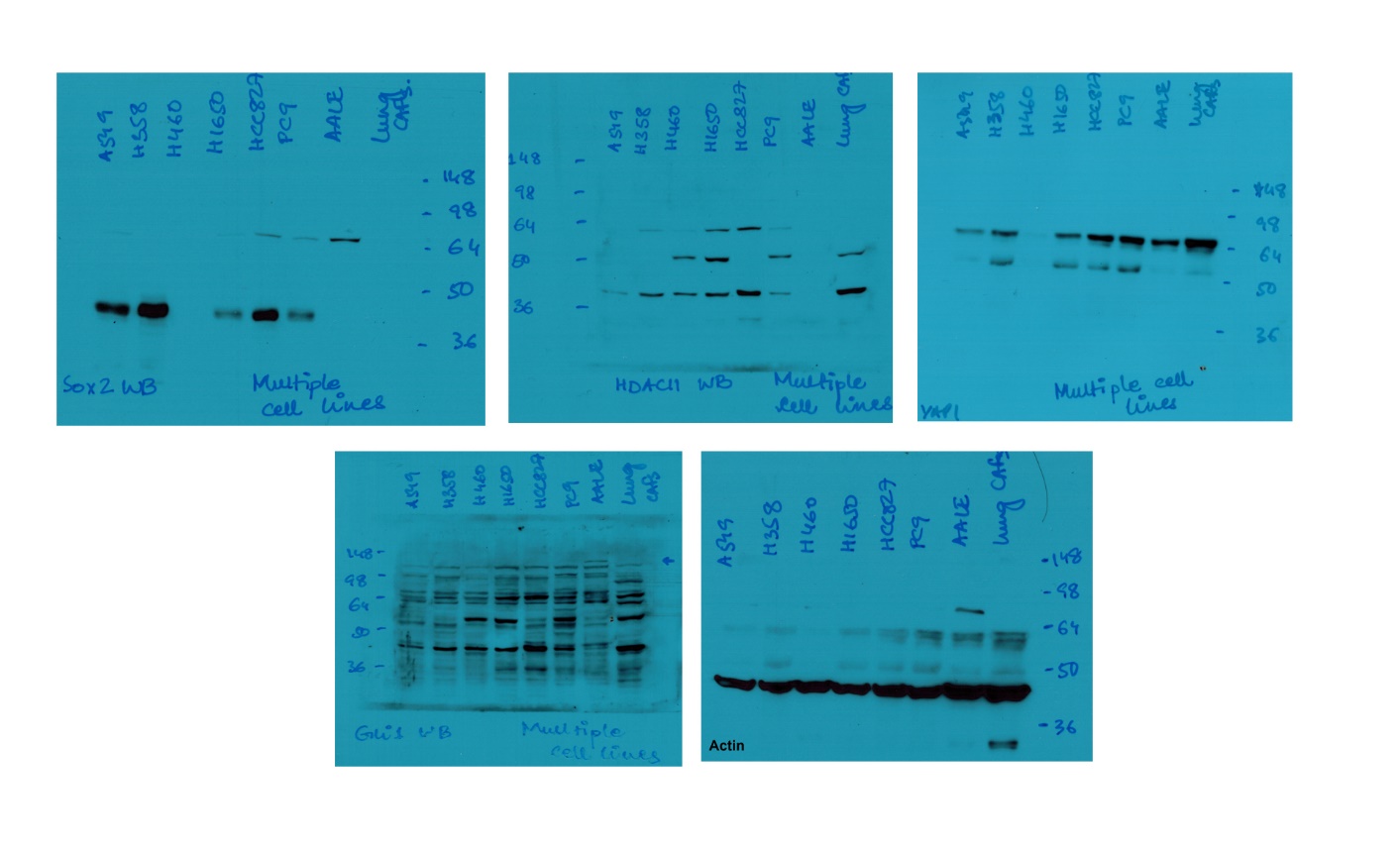


**Supplementary Figure 6.** The images of full length blots of western analysis performed on multiple cell lines presented in the Figure 1F.


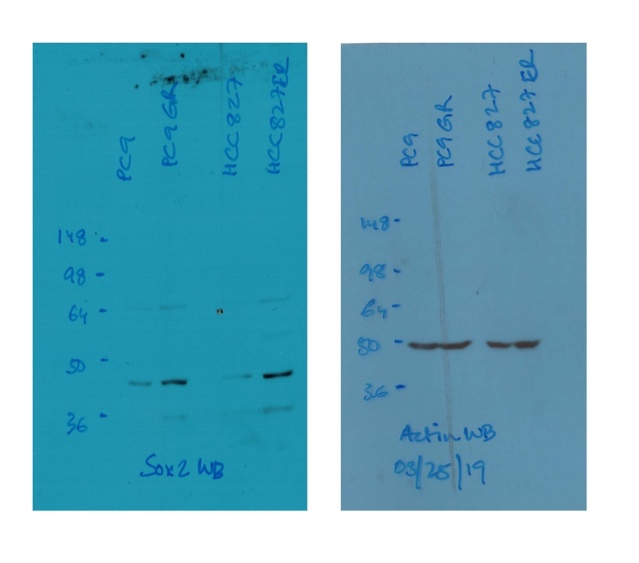


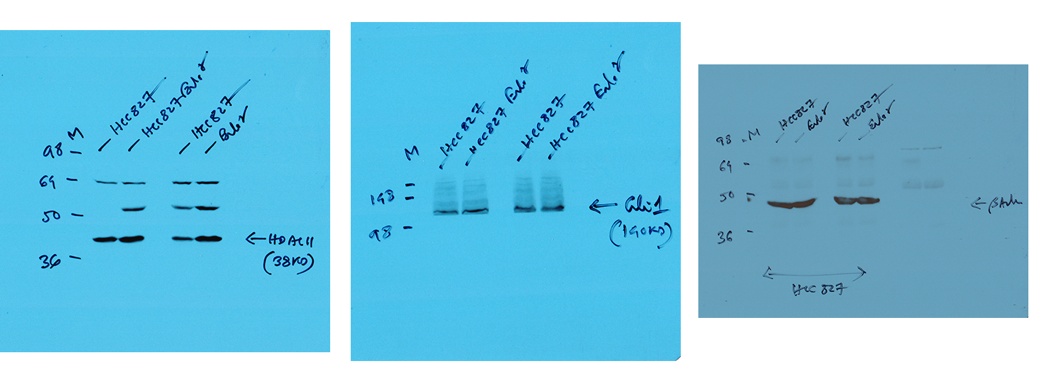


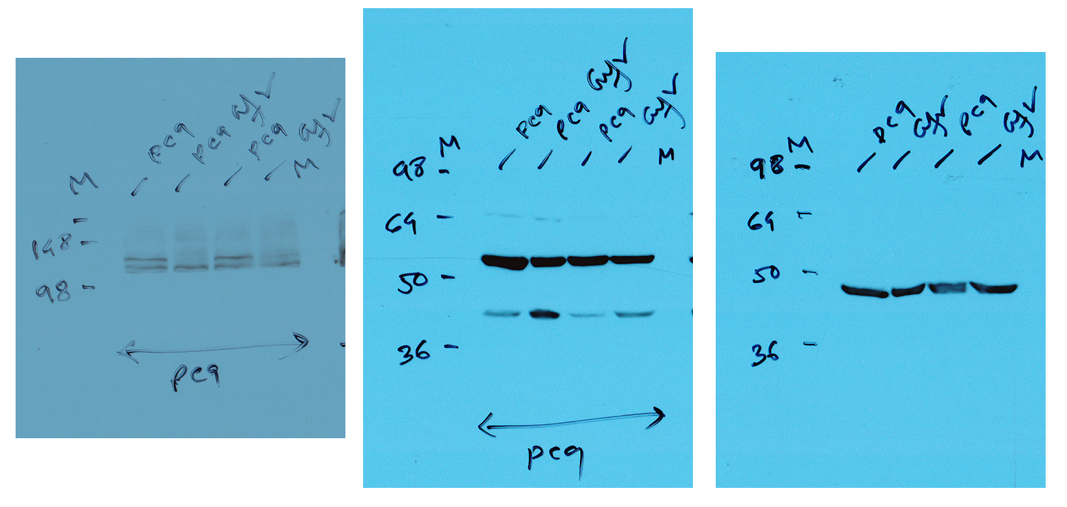


**Supplementary Figure 7.** The images of full length blots of western analysis performed on multiple cell lines presented in the Figure 3B.


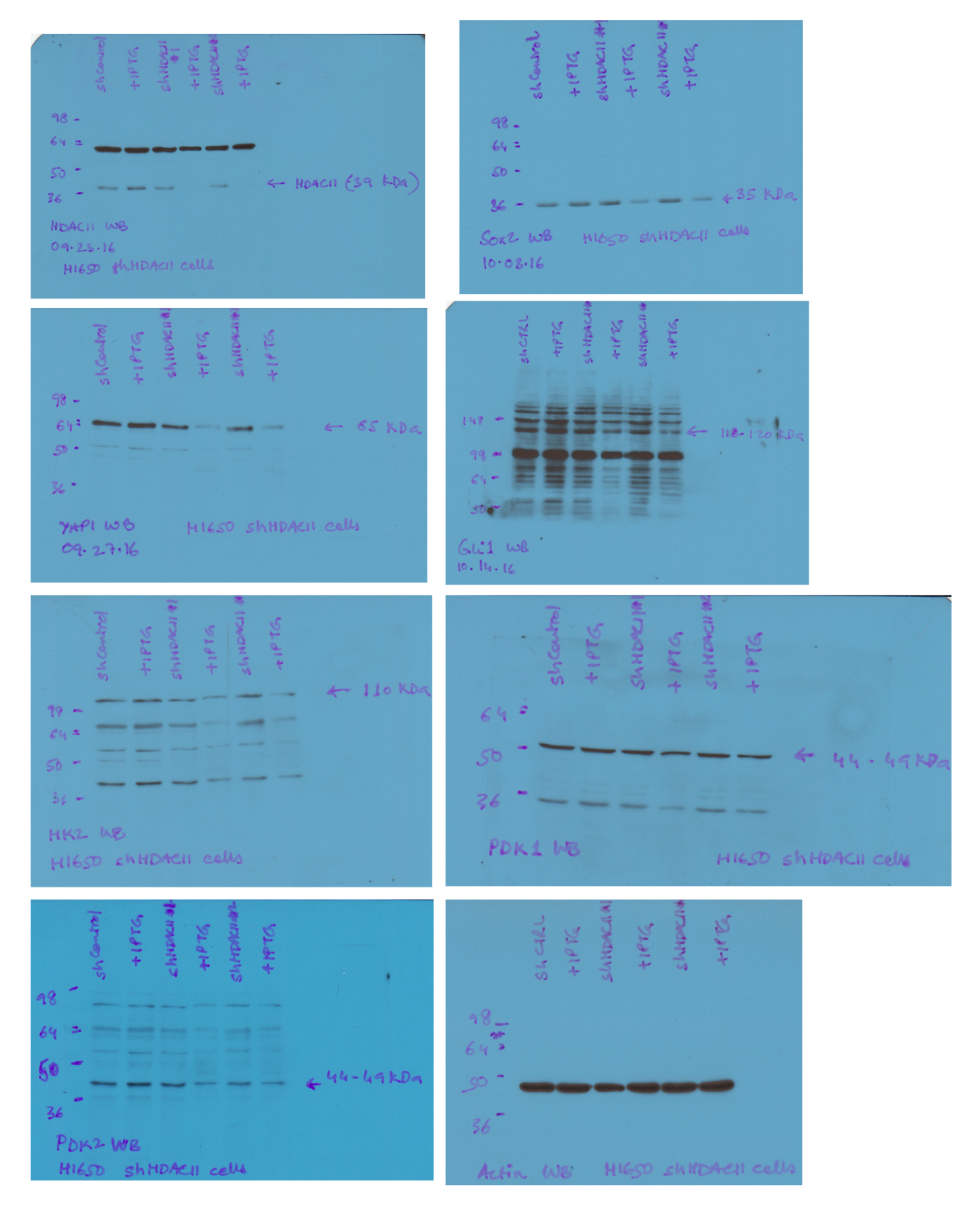


**Supplementary Figure 8.** The images of full length blots of western analysis performed on IPTG inducible HDAC11 knockdown H1650 cells presented in the Figure 4A.


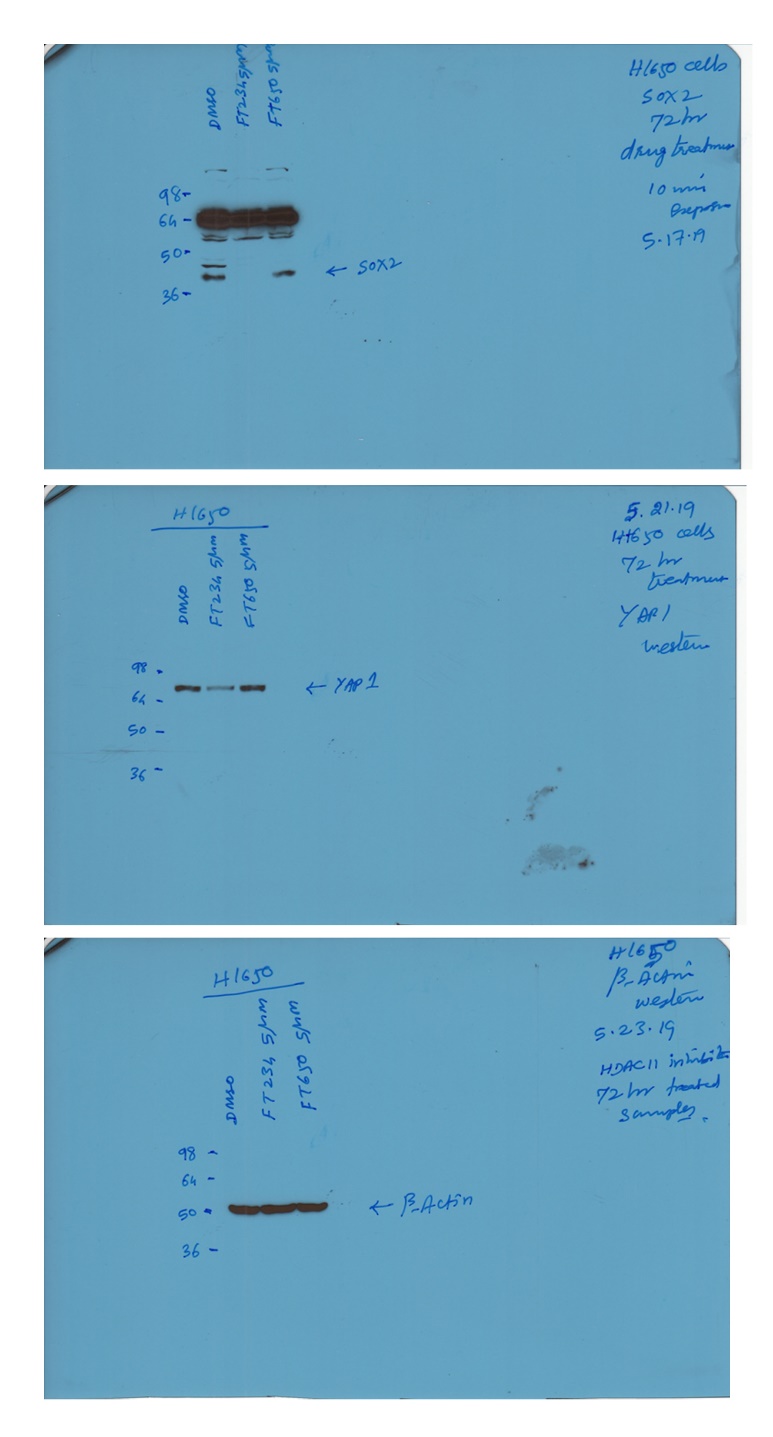


**Supplementary Figure 9.** The images of full length blots of western analysis performed on H1650 cells treated with FT234 and FT650, presented in Figure 7A.

**
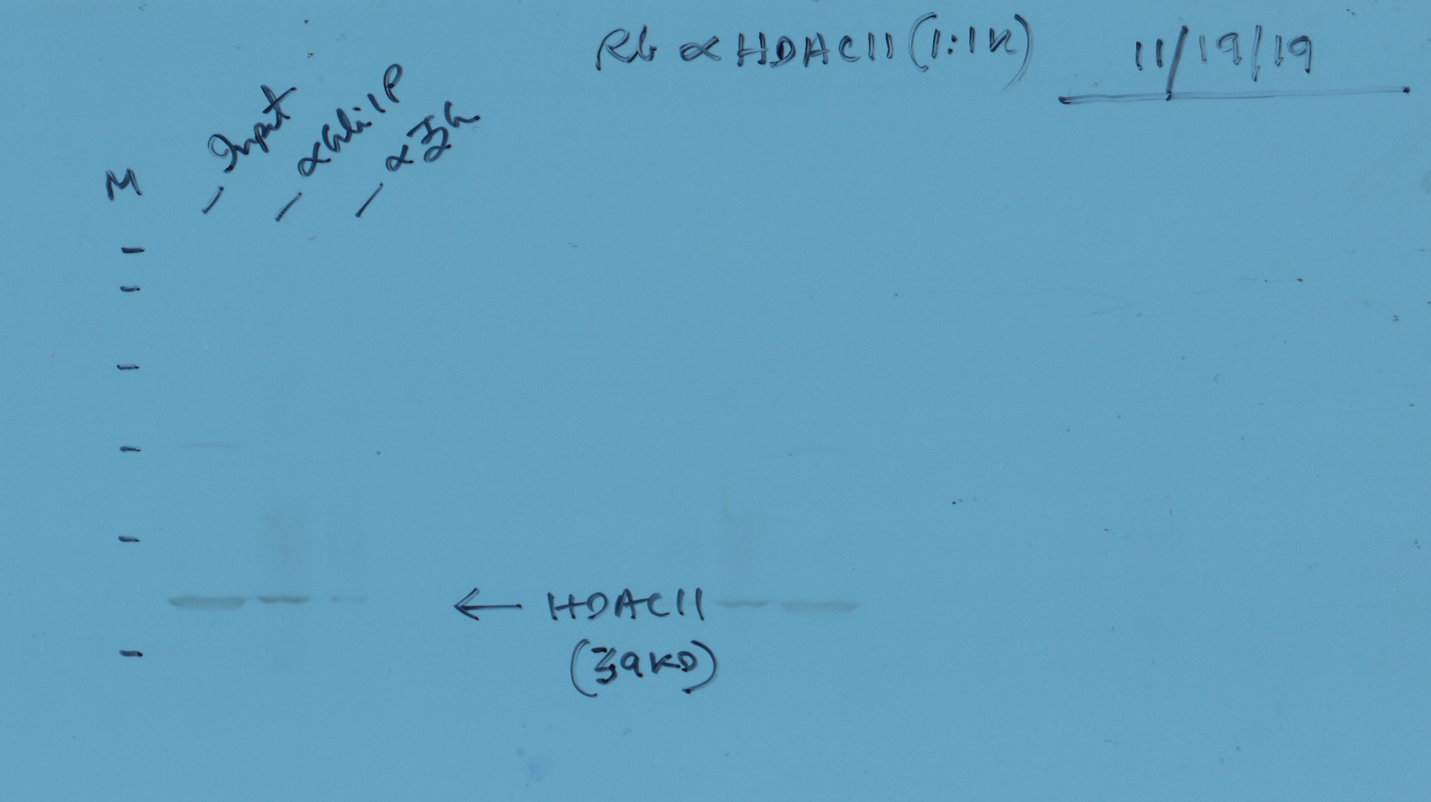
**

**Supplementary Figure 10.** The images of full length blots of IP-western analysis performed on A549 cells (data presented in Supplementary Figure 2).
